# Single‐nucleus transcriptomics reveals subsets of degenerative myonuclei after rotator cuff tear‐induced muscle atrophy

**DOI:** 10.1111/cpr.13763

**Published:** 2024-10-22

**Authors:** Ziying Sun, Xi Cheng, Zheng Wang, Chenfeng Qiao, Hong Qian, Tao Yuan, Zhongyang Lv, Wenshuang Sun, Hanwen Zhang, Yuan Liu, Zhihao Lu, Jintao Lin, Chengteng Lai, Yang Wang, Xiaojiang Yang, Xingyun Wang, Jia Meng, Nirong Bao

**Affiliations:** ^1^ Department of Orthopedics, Nanjing Jinling Hospital, Affiliated Hospital of Medical School Nanjing University Nanjing Jiangsu People's Republic of China; ^2^ Division of Sports Medicine and Adult Reconstructive Surgery, Department of Orthopedic Surgery, Nanjing Drum Tower Hospital, Affiliated Hospital of Medical School Nanjing University Nanjing Jiangsu People's Republic of China; ^3^ State Key Laboratory of Pharmaceutical Biotechnology Nanjing University Nanjing Jiangsu People's Republic of China; ^4^ Department of Orthopedics, Jinling Clinical Medical College Nanjing University of Chinese Medicine Nanjing Jiangsu People's Republic of China; ^5^ Hongqiao International Institute of Medicine, Tongren Hospital Shanghai Jiao Tong University School of Medicine Shanghai People's Republic of China

## Abstract

Rotator cuff tear (RCT) is the primary cause of shoulder pain and disability and frequently trigger muscle degeneration characterised by muscle atrophy, fatty infiltration and fibrosis. Single‐nucleus RNA sequencing (snRNA‐seq) was used to reveal the transcriptional changes in the supraspinatus muscle after RCT. Supraspinatus muscles were obtained from patients with habitual shoulder dislocation (*n* = 3) and RCT (*n* = 3). In response to the RCT, trajectory analysis showed progression from normal myonuclei to ANKRD1^+^ myonuclei, which captured atrophy‐and fatty infiltration‐related regulons (KLF5, KLF10, FOSL1 and BHLHE40). Transcriptomic alterations in fibro/adipogenic progenitors (FAPs) and muscle satellite cells (MuSCs) have also been studied. By predicting cell–cell interactions, we observed communication alterations between myofibers and muscle‐resident cells following RCT. Our findings reveal the plasticity of muscle cells in response to RCT and offer valuable insights into the molecular mechanisms and potential therapeutic targets of RCT.

## Graphical abstract

We performed single‐nucleus RNA‐seq to reveal the transcriptional heterogeneity in myofibers after rotator cuff tear (RCT). Gene signatures of all muscle tissue populations were identified after RCT to reveal the potential transitions of myofibers and other resident cells. Our findings offered a valuable insight for exploring the molecular mechanisms and potential therapeutic targets for RCT.

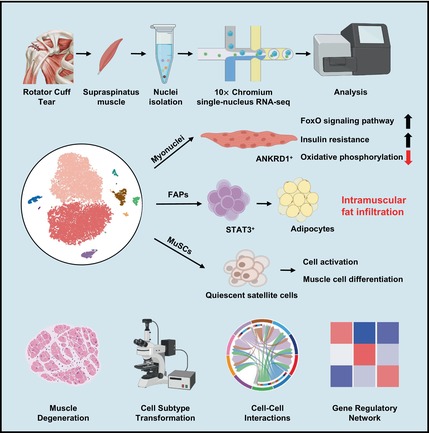

## 1 INTRODUCTION

Rotator cuff tear (RCT) is an increasingly common cause of shoulder pain and disability that eventually requires surgical repair to restore shoulder function.^1^, ^2^, ^3^ Secondary muscle pathologies following RCT include muscle atrophy, fibrosis and fatty infiltration.^4^, ^5^ Despite the performance of reattachment procedures, complete reversal of muscle degeneration remains elusive, leading to tendon tear recurrence and subsequent decline in shoulder function.^6^ The skeletal muscle exhibits great plasticity under pathological conditions, especially in the adaptive response to catabolic stimuli.^7^, ^8^, ^9^, ^10^ Therefore, it is vital to understand the transcriptional landscape of muscle tissues to explore the molecular mechanisms and cell subtype changes after RCT and to develop appropriate treatment strategies.

Myofibers are characterised by their large size and multiple myonuclei, they are typically categorised into type I and type II myofibers. Type II myofibers can be further classified as type IIa, IIx or IIb.^11^, ^12^ The spaces between these fibres contain various muscle‐resident cells, including fibro/adipogenic progenitors (FAPs), muscle satellite cells (MuSCs), endothelial cells and immune cells such as macrophages and lymphocytes.^13^ Balancing these diverse cell populations is essential for maintaining muscle function and homeostasis.^14^, ^15^ To achieve this, the specific gene regulatory networks (GRNs) initiate varied gene expression patterns in each cell type, resulting in the complex transcriptional heterogeneity of the tissue.

Single‐cell RNA sequencing (scRNA‐seq) enables the simultaneous detection of cell subtypes in tissues in both physiological and pathological states. It has been used to characterise the gene expression profiles of several types of tissues, including cartilage, synovium and muscle.^16^, ^17^ However, a thorough examination of the transcriptional heterogeneity within multinucleated tissues has proven challenging, primarily because of the shared cytoplasmic environment. With advances in databases and sequencing technology, single‐nucleus RNA sequencing (snRNA‐seq) has been used to detect transcriptional states in multinucleated cells such as myofibers.^18^, ^19^, ^20^, ^21^ Recent studies have revealed that the myonuclei within syncytial muscle fibres possess distinct transcriptional profiles that regulate muscle biology. However, the complete gene expression landscape of myofibers and muscle‐resident cells after RCT remains unclear.

In this study, snRNA‐seq was used to reveal the transcriptional heterogeneity in myofibers after RCT. We chose the supraspinatus muscle because it is the most often injured in RCT.^22^ The gene signatures of all muscle tissue populations were identified after RCT to reveal the potential transitions of myofibers and other resident cells. Furthermore, the potential cell communication was evaluated, providing a comprehensive single‐nucleus atlas of the supraspinatus muscle after RCT. These results provide novel strategies for the treatment of RCT.

## 2 RESULTS

### 2.1 The heterogeneity of muscle and cellular composition in both normal and RCT groups

**FIGURE 1:**
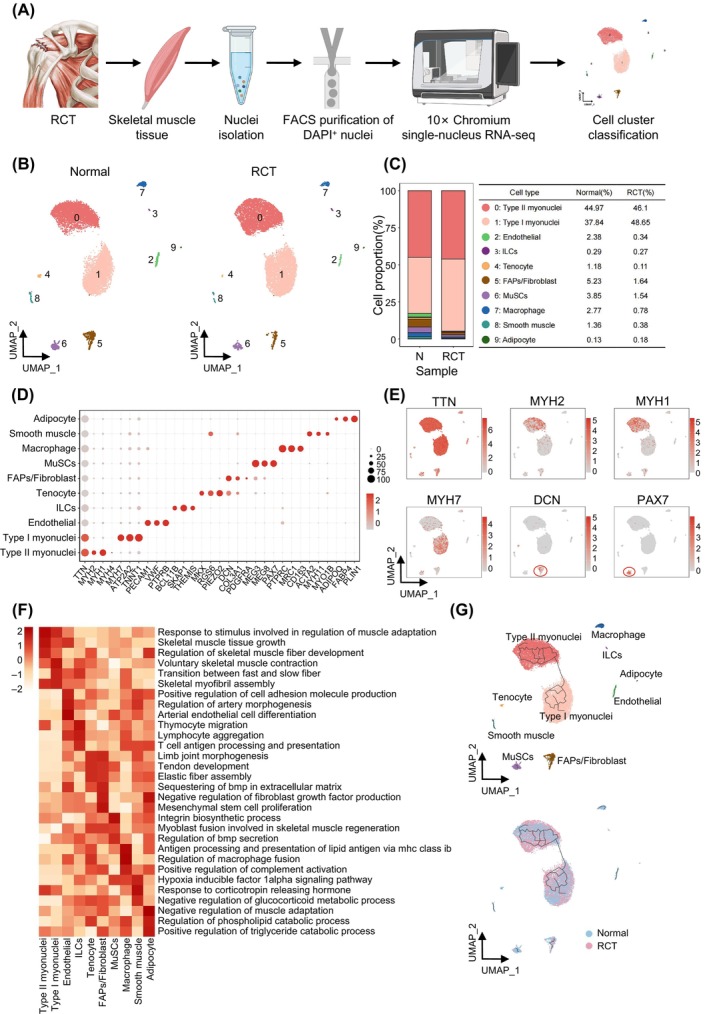
Classification of nucleus/cell types of supraspinatus muscles in normal individuals and patients with rotator cuff tear (RCT). (A) Schematic diagram of nuclei purification and sequencing for human supraspinatus muscles. (B) Uniform manifold approximation and projection (UMAP) was performed to visualise nuclear clusters, which are coloured and labelled based on cell identities, in normal (left panel) and RCT (right panel) supraspinatus muscles. 0, type II myonuclei; 1, type I myonuclei; 2, endothelial cells nuclei (endothelial); 3, innate lymphoid cells nuclei (ILCs); 4, tenocytes nuclei (tenocytes); 5, fibro/adipogenic progenitor nuclei (FAPs/fibroblasts); 6, muscle satellite cells nuclei (MuSCs); 7, macrophages nuclei (macrophages); 8, smooth muscle cells nuclei (smooth muscle) and 9, adipocytes nuclei (adipocytes). (C) Proportion of nuclear types in normal and RCT supraspinatus muscles. Each nucleus/cell type is colour‐coded. (D) Dot plot showing the expression of cell identities in each nuclear cluster. Dot sizes represent the percentages of nuclei expressing a gene. Colour intensity of dots indicate gene expression level; colour scale indicates expression level. (E) UMAP plot showing the cell identities of specific nuclear clusters, including TTN, MYH2, MYH1, MYH7, DCN and PAX7. (F) Gene set variation analysis (GSVA) of each cell type in supraspinatus muscles. Colour scale indicates the enrichment level. (G) UMAP plots with pseudotime trajectories of all nuclei obtained from normal and RCT supraspinatus muscles. Black lines on the UMAP plots represent branched trajectories. Each point denotes a single nucleus. Upper panel, nuclei are colour‐coded according to their cluster assignments in (B); lower panel, nuclei are coloured based on conditions (blue, normal; pink, RCT).

To investigate the heterogeneity of muscle changes after RCT, the supraspinatus muscles from three patients with habitual shoulder dislocation and three patients with RCT were obtained (Figure 1A, Figure S1A). Isolated muscle nuclei were used to generate single‐nucleus libraries using the 10× Genomics Chromium System. Following rigorous quality control procedures, 16,893 nuclei were retained, comprising 8990 nuclei from patients in the normal group and 7903 nuclei from the RCT group, respectively. Nineteen unsupervised clusters were identified using the Seurat package (Figure S1B,C). Based on specific gene expression patterns, all clusters were categorised into 10 distinct cell types: type II myonuclei, type I myonuclei, endothelial cells, innate lymphoid cells (ILCs), tenocytes, FAPs/fibroblasts, MuSCs, macrophages, smooth muscle cells and adipocytes (Figure 1B). First, we examined changes in cell populations and observed that the normal supraspinatus muscle predominantly consisted of type II (44.97%) and type I myonuclei (37.84%). Conversely, in response to RCT, there was an increase in the proportion of type II (46.1%) and type I myonuclei (48.65%) (Figure 1C). The top signature genes in each cell type are shown in Figure 1D and Figure S1D. Feature plots of lineage‐specific markers were shown in Figure 1E and Figure S1E, including TTN, MYH2, NYH1 and MYH7.^23^ To further elucidate the heterogeneity of the supraspinatus muscles, gene set variation analysis (GSVA) of gene ontology (GO) pathways was conducted for each cell type. Notably, type II myonuclei were enriched in response to stimuli involved in the regulation of muscle adaptation, whereas type I myonuclei were enriched during the transition between fast and slow fibres. Interestingly, adipocytes were enriched in negatively regulated muscle adaptation, suggesting their potential role in muscle transition in response to stimulation (Figure 1F).

Subsequently, a pseudotime analysis of all nuclei post‐RCT was conducted using Monocle3. Trajectories were constructed based on transcriptional similarities, providing insights into the dynamic changes in the expression profiles of various cell types. As shown in Figure 1G, the most dynamic trajectories were observed in type II myonuclei, followed by type I myonuclei, and FAPs/fibroblasts. This suggests that RCT induces more heterogeneous and transcriptional alterations in the myonuclei and FAPs.

### 2.2 The heterogeneity of type II myonuclei following RCT

**FIGURE 2:**
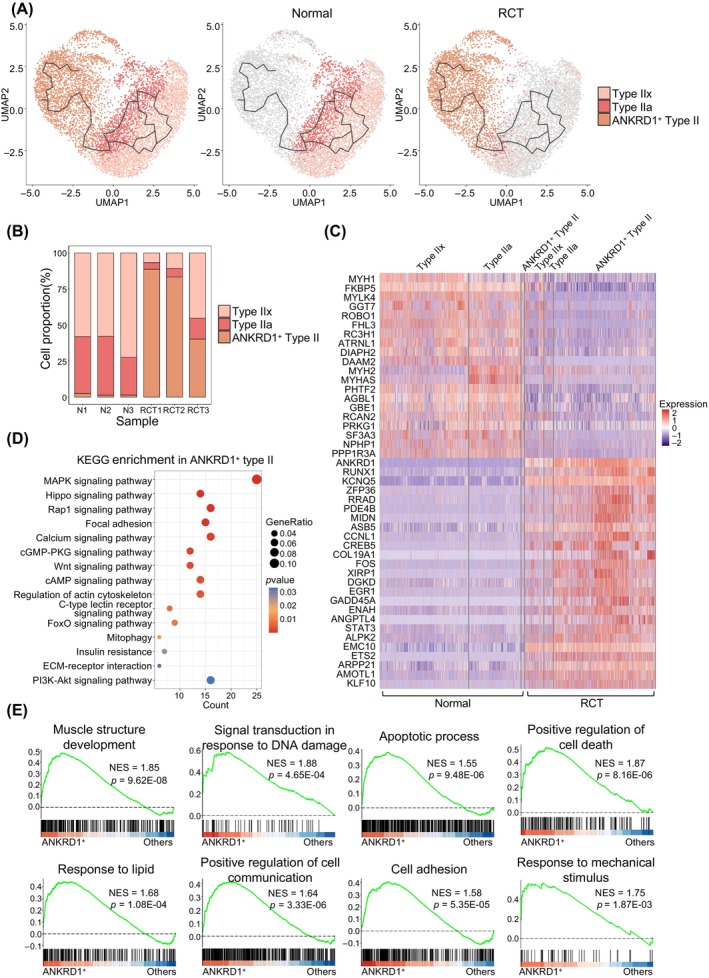
Trajectory‐illustrated transcriptional heterogeneity in type II myonuclei. (A) Uniform manifold approximation and projection (UMAP) showing trajectory of type II myonuclei from normal (middle panel, *n* = 4043) and rotator cuff tear (RCT) (right panel, *n* = 3643) supraspinatus muscles. Myonuclei are coloured based on their identified subtypes. Type IIx, type IIx myonuclei; type IIa, type IIa myonuclei; and ANKRD1^+^ type II, ANKRD1^+^ type II myonuclei. (B) Proportions of different type II myonuclei subtypes under normal and RCT conditions. (C) Heatmap showing differentially expressed genes (DEGs) of type II myonuclei subtypes. Colour scale represents the relative expression levels of genes in each nucleus. (D) Enriched Kyoto Encyclopaedia of Genes and Genomes (KEGG) pathways (*p* < 0.05) in ANKRD1^+^ type II myonuclei. Colour scale indicates the significance level of enrichment (*p* value). Dot size represents gene ratio in the pathway. (E) Gene set enrichment analysis (GSEA) plots showing enrichment score (ES) of the significant enriched gene ontology biological process (GOBP) gene sets in ANKRD1^+^ type II myonuclei. A positive value of ES indicates genes enriched in ANKRD1^+^ type II myonuclei but down‐regulated in others.

To elucidate the response of type II myonuclei to RCT, we first divided all type II myonuclei into three subtypes, type IIx myonuclei, type IIa myonuclei, and ANKRD1^+^ type II myonuclei, using the Seurat package (Figure 2A, left panel). Consistent with a previous study,^24^ type IIb myonuclei were not included in the supraspinatus muscle, which is characterised by MYH4 (Figure S2A). Monocle3 results showed complex trajectories from type IIx and type IIa myonuclei in the normal group (Figure 2A, middle panel) to ANKRD1^+^ type II myonuclei in the RCT group (Figure 2A, right panel). These trajectories were accompanied by an increased proportion of ANKRD1^+^ type II myonuclei (Figure 2B), signifying a transition from normal to specialised myonuclei. To characterise type II myonuclei, differentially expressed genes (DEGs) for different cell subtypes were investigated in the normal and RCT groups. Normal type IIx and type IIa myonuclei were identified using MYH1 and MYH2, respectively, and were down‐regulated in the RCT group (Figure 2C, Figure S2A). In response to RCT, ANKRD1^+^ type II myonuclei showed higher expression of several atrophy‐associated genes, such as RUNX1, GADD45A, STAT3 and PDE4B (Figure 2C, Figures S2B,C).^25^, ^26^ Kyoto Encyclopedia of Genes and Genomes (KEGG) enrichment analysis demonstrated that MAPK, Hippo and Rap1 signalling pathways were enriched in ANKRD1^+^ type II myonuclei (Figure 2D). GO enrichment analysis showed that muscle cell differentiation, skeletal muscle adaptation, and positive regulation of proteasomal protein catabolic processes were enriched only in the ANKRD1^+^ type II myonuclei (Figure S3A–C). Gene set enrichment analysis (GSEA) of GO revealed that biological process (BP)‐associated gene related to apoptosis process, positive regulation of cell death, response to lipid were enriched in ANKRD1^+^ type II myonuclei (Figure 2E). Conversely, BP‐associated gene sets involving carbohydrate catabolic processes, cellular respiration and ATP metabolic processes were negatively correlated with ANKRD1^+^ type II myonuclei (Figure S3D). The GSEA results suggest changes in the metabolic demand for type II myonuclei.

### 2.3 The heterogeneity alterations of type I myonuclei in response to RCT

**FIGURE 3:**
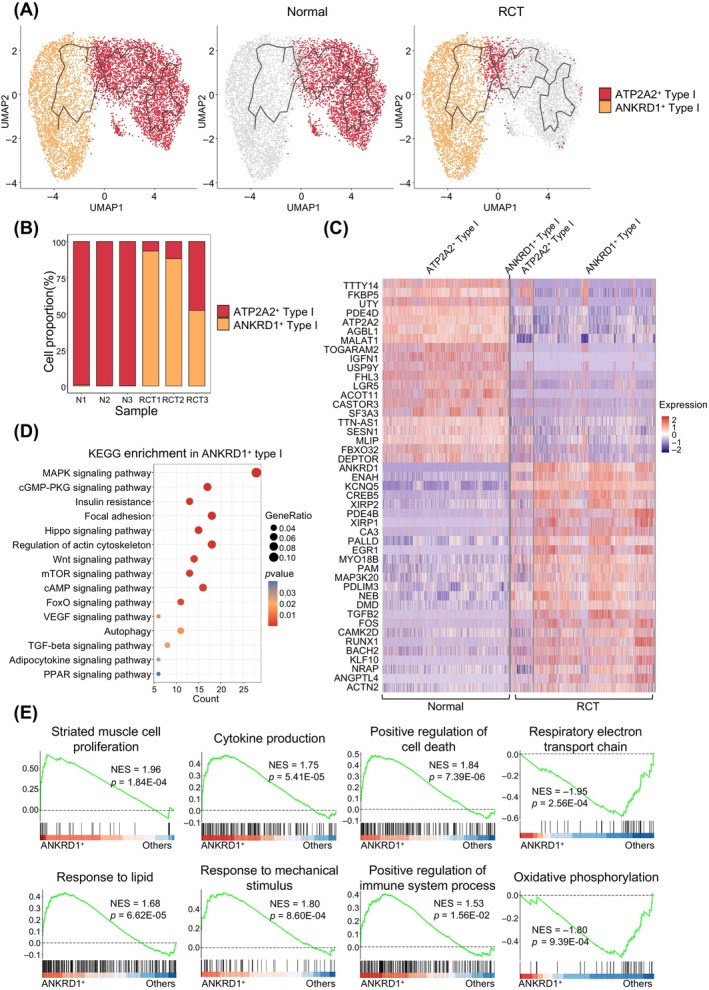
Trajectory‐illustrated transcriptional heterogeneity in type I myonuclei. (A) Uniform manifold approximation and projection (UMAP) showing trajectory of type I myonuclei from normal (middle panel, *n* = 3402) and rotator cuff tear (RCT) (right panel, *n* = 3845) muscles. The myonuclei are coloured based on their identified subtypes. ATP2A2^+^ type I, ATP2A2^+^ type I myonuclei; and ANKRD1^+^ type I, ANKRD1^+^ type I myonuclei. (B) Proportions of different type I myonuclei subtypes under normal and RCT conditions. (C) Heatmap showing differentially expressed genes (DEGs) of type I myonuclei subtypes. Colour scale represents the relative expression levels of genes in each nucleus. (D) Enriched Kyoto Encyclopedia of Genes and Genomes (KEGG) pathways (*p* < 0.05) in ANKRD1^+^ type I myonuclei. Colour scale indicates the significance level of enrichment (*p* value). Dot sizes represent gene ratios in the pathway. (E) Gene set enrichment analysis (GSEA) plots showing enrichment score (ES) of the significantly enriched gene ontology biological process (GOBP) gene sets in ANKRD1^+^ type I myonuclei. A positive ES value indicates genes enriched in ANKRD1^+^ type I myonuclei, and a negative value indicates genes enriched in other ANKRD1^+^ type I myonuclei but down‐regulated in other ANKRD1^+^ myonuclei.

Unlike type II myofibers, previous studies have identified fewer subtypes for type I myofibers.^11^, ^12^ To uncover alterations in type I myonuclei in response to RCT, we initially classified all type I myonuclei into two subtypes, ATP2A2^+^ type I myonuclei and ANKRD1^+^ type I myonuclei, using Seurat (Figure 3A, left panel). Subsequently, we constructed the trajectory of type I myonuclei using Monocle3. The results revealed trajectories progressing from ATP2A2^+^ type I myonuclei in the normal group (Figure 3A, middle panel) to ANKRD1^+^ type I myonuclei in the RCT group (Figure 3A, right panel). The proportion of cell subtypes also indicated an increased proportion of ANKRD1^+^ type I myonuclei (Figure 3B).

The top genes were identified through DEGs analysis, with TTTY14, FKBP5, UTY and PDE4D being highly expressed in ATP2A2^+^ type I myonuclei, and KCNQ5, CREB5, XIRP2 and CA3 being highly expressed in ANKRD1^+^ type I myonuclei (Figure 3C). KEGG analysis revealed enrichment in the MAPK signalling pathway, cGMP‐PKG pathway and insulin resistance in ANKRD1^+^ type I myonuclei (Figure 3D). GO enrichment results for BP demonstrated enrichment in muscle cell differentiation, muscle contraction, regulation of calcium ion transport and regulation of proteasomal ubiquitin‐dependent protein catabolic process in ANKRD1^+^ type I myonuclei (Figure S4A,B). GSEA of the BP results revealed that gene sets associated with cytokine production, positive regulation of cell death, response to lipid and response to mechanical stimulus were enriched in the ANKRD1^+^ type I myonuclei (Figure 3E). Conversely, NADP metabolic process, aerobic respiration and generation of precursor metabolic and energy were enriched in ATP2A^+^ type I myonuclei (Figure S4C).

### 2.4 Transcriptomic changes in type II and type I myonuclei after RCT

**FIGURE 4:**
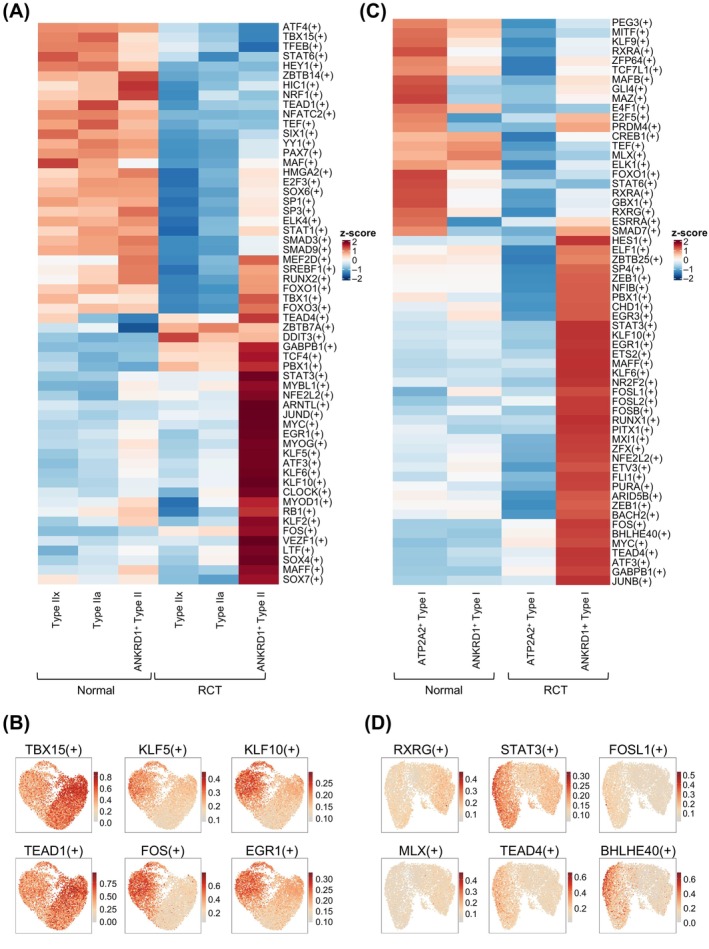
Gene regulatory networks (GRNs) of myonuclei in the normal and rotator cuff tear (RCT) groups. (A) Heatmap showing regulons with significant changes between normal and RCT type II myonuclei. Colour scale represents the activities of regulons. Significant changed regulons are presented on the right. (B) Uniform manifold approximation and projection (UMAP) showing that RCT suppresses (left panel) or enhances (right panel) the activities of selected regulons in type II myonuclei. (C) Heatmap showing regulons with statistically significant changes between normal and RCT type I myonuclei. Colour scale represents the activities of regulons. Significantly changed regulons are presented on the right. (D) UMAP showing that RCT suppresses (left panel) or enhances (right panel) the activities of selected regulons in type I myonuclei.

Single‐cell regulatory network inference and clustering (SCENIC) was performed to investigate the activity of GRNs in type II and type I myonuclei in both the normal and RCT groups. Regulons that potentially mediate transcriptomic and phenotypic changes in type II myonucleus subtypes in specific states were identified (Figure 4A). The feature plot results further supported the finding that RCT significantly altered regulon expression in type II myonuclei. For instance, TBX15 and TEAD were active in nearly all type II myonuclei from the normal group, whereas KLF5, KLF10, FOS and EGR1 were specifically active in ANKRD1^+^ type II myonuclei after RCT (Figure 4B). KLF5 and KLF10 are key mediators of early muscle atrophy,^27^, ^28^ whereas FOS increases after denervation‐induced muscle atrophy.^29^

In Figure 4C,D, alterations in the regulons of type I myonuclei in response to RCT are shown. Our results suggested that transcription factors STAT3, FOSL1, BHLHE40, which promote degenerative changes in muscle atrophy and intramuscular fat infiltration, are up‐regulated.^29^, ^30^ Some transcription factors such as PEG3, KLF9, STAT6 and RXRA were inactivated after RCT. These findings highlight the activation of specific regulons in the RCT group, suggesting their potential role in driving transcriptional changes.

### 2.5 Heterogeneity and phenotype of FAPs after RCT

**FIGURE 5:**
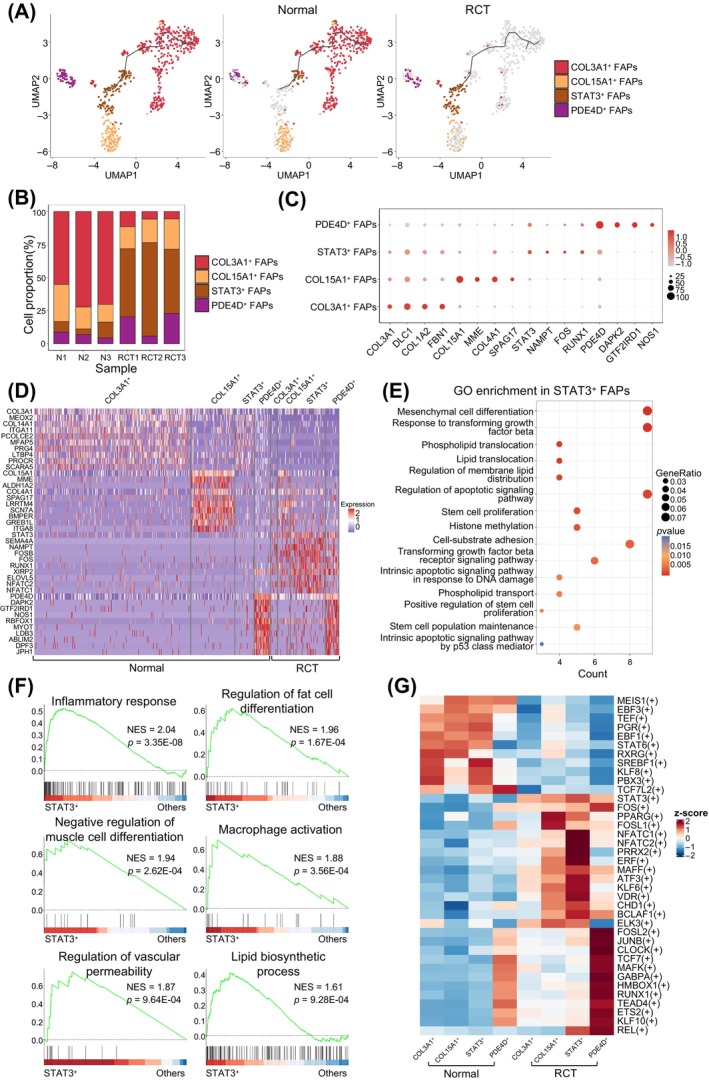
Transcriptional responses to rotator cuff tear (RCT) in fibro/adipogenic progenitors (FAPs). (A) Uniform manifold approximation and projection (UMAP) showing a branching trajectory of FAPs from normal muscles (middle panel, *n* = 470) and RCT muscles (right panel, *n* = 130). The nuclei mapped on the path are coloured and labelled according to their identified subtypes as COL3A1^+^, COL15A1^+^, STAT3^+^ and PDE4D^+^ FAPs. (B) Changes in proportions of FAPs subtypes in normal and RCT muscles. Different colours represent the corresponding subtypes. (C) Dot plot displaying the average expression level of FAPs identities. Dot size represents the percentage of FAPs expressing a gene within the subtype. The colour intensity of the dot indicates gene expression level and the colour scale indicates the expression level. (D) Heatmap displaying the top 10 differentially expressed genes (DEGs) of identified FAPs subtypes. The colour scale represents the relative expression level of gene in each kind of nucleus. (E) Enriched gene ontology biological process (GOBP) pathways (*p* < 0.05) in STAT3^+^ FAPs. The colour scale indicates the significance level of enrichment (*p* value). Dot size represents gene ratio in the pathway. (F) Gene set enrichment analysis (GSEA) plots showing enrichment score (ES) of the significant enriched GOBP gene sets in STAT3^+^ FAPs. A positive value of ES indicates enriched in STAT3^+^ FAPs but down‐regulated in others. (G) Heatmap showing the changes in activity scores of regulons in FAPs subtypes between normal and RCT groups. The colour scale represents the normalised scores of regulon activity: red indicates high level of activity and blue indicates low level of activity.

To explore the transcriptomic changes in FAPs in response to RCT, FAPs were categorised into four subtypes: COL3A1^+^ FAPs, COL15A1^+^ FAPs, STAT3^+^ FAPs and PDE4D^+^ FAPs (Figure 5A, left panel). The trajectory of the FAPs was reconstructed, revealing fewer nodes and branches than in type I and II myonuclei. Trajectory analysis revealed a transition from COL3A1^+^ and COL15^+^ FAPs in the normal group (Figure 5A, middle panel) to STAT3^+^ FAPs in the RCT group (Figure 5A, right panel). The cell proportion analysis confirmed these changes (Figure 5B). DEGs analysis revealed heterogeneity changes in FAPs after RCT, with COL3A1^+^ FAPs showing higher expression of COL3A1, DLC1, COL1A2 and FBN1; COL15A1^+^ FAPs exhibiting higher expression of COL15A1, MME, COL4A1 and SPAG17; STAT3^+^ FAPs displaying high expression of STAT3, NAMPT, FOS and RUNX1, these genes were shown to promote intramuscular fat production^30^, ^31^; and PDE4D^+^ FAPs having higher expression of PDE4D, DAPK2 and NOS1, indicating diverse responses to RCT (Figure 5C,D).

GO analysis revealed the enrichment of mesenchymal cell differentiation, response to transforming growth factor‐beta, and phospholipid translocation in STAT3^+^ FAPs (Figure 5E). COL3A1^+^ and COL15A1^+^ FAPs were enriched in extracellular matrix organisation and collagen‐related processes (Figure S5A,B). PDE4D^+^ FAPs were enriched in muscle atrophy, regulation of lipid localization and regulation of lipid transport (Figure S5C). GSEA demonstrated that the regulation of fat cell differentiation, negative regulation of muscle cell differentiation and lipid biosynthetic processes were enriched in STAT3^+^ FAPs. In addition, inflammation‐related gene sets such as inflammatory response, macrophage activation and regulation of vascular permeability were enriched, indicating that FAPs participate in the pro‐inflammatory process after RCT (Figure 5F).

As shown in Figure 5G, SCENIC analysis was performed to investigate regulons whose activities were significantly changed by RCT in FAPs. A list of master regulons that may mediate phenotypic changes in FAPs is provided to illustrate the regulon changes during cell subtype alterations. For instance, PPARG, ATF3, KLF6 and NFATC1 regulons were enhanced in STAT3^+^ FAPs after the RCT, whereas TCF7, MAFK and RUNX1 regulons were activated in PDE4D^+^ FAPs in the RCT group. PPARG, KLF6 and RUNX1 promote intramuscular fat production.^31^, ^32^, ^33^

### 2.6 Alterations in expression profile of MuSCs in response to RCT

**FIGURE 6:**
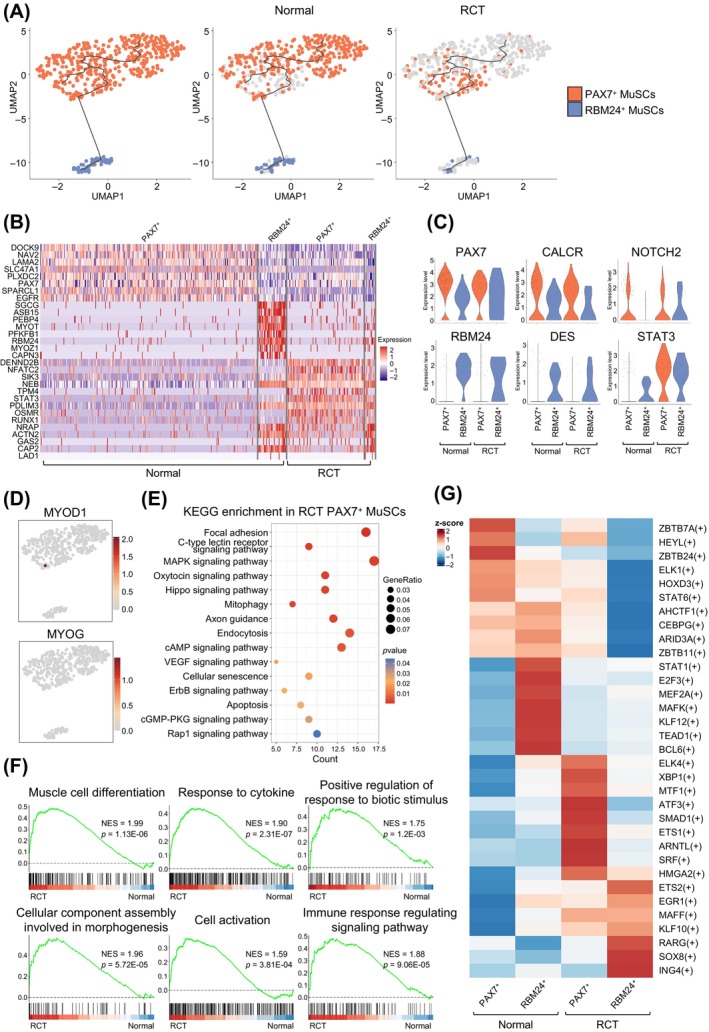
The transcriptome and heterogeneity of muscle satellite cells (MuSCs) within rotator cuff tear (RCT) muscle. (A) Uniform manifold approximation and projection (UMAP) showing a branching trajectory of MuSCs from normal muscles (middle panel, *n* = 346) and RCT muscles (right panel, *n* = 122). The nuclei mapped on the path are coloured and labelled according to their identified subtypes as PAX7^+^ and RBM24^+^ MuSCs. (B) Heatmap showing differentially expressed genes (DEGs) between normal and RCT MuSCs. The colour scale represents the relative level of gene expression: dark blue indicates low level and red indicates high level of expression. (C) Violin plot showing comparison between normal and RCT gene expression in different MuSCs subtypes. (D) Visualisation of the expression of MYOD1 and MYOG, which are markers of myoblasts. (E) Significantly enriched Kyoto Encyclopedia of Genes and Genomes (KEGG) pathways (*p* < 0.05) in RCT PAX7^+^ MuSCs. The colour scale indicates the significance level of enrichment (*p* value). Dot size represents gene ratio in the pathway. (F) Gene set enrichment analysis (GSEA) plots showing ES of the significant enriched GOBP gene sets in RCT MuSCs. A positive value of enrichment score (ES) indicates enrichment in the RCT group but down‐regulated in the normal group. (G) Heatmap showing significant changed regulons between normal and RCT MuSCs. The colour scale represents the normalised scores of regulon activity: dark blue indicates low level of activity and red indicates high level of activity.

In response to injury, quiescent MuSCs activate, transition to myoblasts and differentiate into myocytes, which fuse and regenerate multinucleate myofibers.^34^, ^35^ Initially, we classified MuSCs into two subtypes: PAX7^+^ MuSCs and RBM24^+^ MuSCs (Figure 6A, left panel). Trajectories were constructed to reveal potential phenotypic alterations that demonstrated no significant differences between the normal and RCT groups (Figure 6A, middle and right panels, respectively). Cell proportion analysis of the two cell types showed no discernible changes (Figure S6A). CALCR and NOTCH2, which are associated with the resting state of MuSCs, exhibit decreased expression after RCT.^36^, ^37^, ^38^, ^39^ Conversely, activation‐related genes such as DES and STAT3 showed an increased expression (Figure 6B,C).^40^, ^41^ Interestingly, we detected low expression of the myogenic commitment factors MYOD1 and MYOG (Figure 6D), in contrast to the scRNA‐seq analyses of adult mouse muscle, suggesting that MuSCs are composed largely of muscle stem cells and not committed myogenic progenitors (myoblasts).^42^, ^43^ KEGG analysis revealed that PAX7^+^ MuSCs were enriched in focal adhesion, C‐type lectin receptor signalling pathway, and MAPK signalling pathway in the RCT group (Figure 6E). In addition, GO analysis revealed enhanced muscle cell differentiation, myofiber assembly, and the ERBB signalling pathway in PAX7^+^ MuSCs after RCT (Figure S6B). GSEA demonstrated that gene sets related to cellular stress changes, such as response to cytokine, cell activation and immune response‐regulating signalling pathways, were significantly enhanced in RCT PAX7^+^ MuSCs (Figure 6F).

Subsequently, SCENIC analysis was performed to explore the underlying mechanisms of the phenotypic changes in MuSCs. Distinct patterns of regulon activity were observed in all cell subtypes, including ZBTB7A, HEYL and STAT6 in PAX7^+^ MuSCs and STAT1, E2F3 and MEF2A in RBM24^+^ MuSCs in the normal state (Figure 6G). Notably, these regulons were found to be inactive in the RCT group. Previous studies have shown that HEYL and STAT6 are beneficial for maintaining the undifferentiated and quiescent states of MuSCs in their niche.^44^ Simultaneously, XBP1, ATF3, ARNTL and SMAD1 in PAX7^+^ MuSCs and ETS2, RARG and EGR1 in RBM24^+^ MuSCs were significantly activated in the RCT group (Figure 6G). These regulons stimulated MuSC proliferation and prevented precocious myogenic differentiation.^45^, ^46^, ^47^, ^48^ Taken together, our results suggest that MuSCs in the RCT group are in a stage of early activation, proliferation, and myogenesis.

### 2.7 Interactions between myofibers and resident cells in response to RCT

**FIGURE 7:**
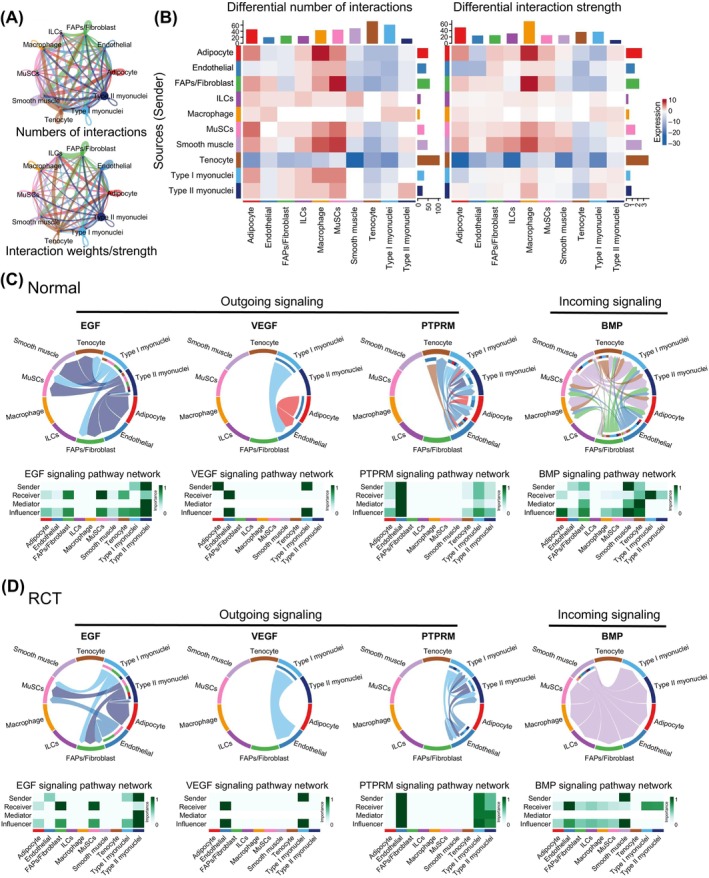
Ligand‐receptor interactions between myofibers and other resident cells in response to rotator cuff tear (RCT). (A) Circle plots showing the numbers (upper panel) and strengths (lower panel) of intercellular ligand‐receptor (L‐R) interactions in supraspinatus muscles between normal and RCT group. The thicker the line represented, the more the numbers of interactions, and the stronger the interaction weights/strength between the two cell types. (B) Heatmap displaying the differences in the number of interactions (left panel) and the interaction strength (right panel) between normal group and RCT group. Dark blue indicates lower level of interactions and red indicates higher level of interactions of RCT group. (C and D) Chord diagram inferred the EGF, VEGF and PRPRM myofiber outgoing signalling and BMP myofiber incoming signalling in normal (C) and RCT (D) muscles with CellChat. The size of the width of various colours in the periphery indicates probability/intensity value of interaction (intensity is the sum of probability values). Heatmaps quantify the role of each cluster as a sender, receiver, mediator and influencer.

To investigate the cell communication between myofibers and other resident cells in response to RCT, CellChat was used to calculate the interactions between myonuclei and resident cells. Based on the interaction numbers and weights, the interaction networks illustrated complex regulatory effects on other cell types for all cell types, except ILCs and macrophages (Figure 7A, Figure S7A). Differential interaction numbers and strengths were also analysed between the normal and RCT groups. The interaction numbers of type I and type II myonuclei with most resident cells were enhanced in the RCT group, indicating tear‐induced transcriptomic changes. Interestingly, interactions from adipocytes and FAPs to macrophages and MuSCs were significantly enhanced, potentially revealing alterations in cell communication under RCT conditions (Figure 7B).

The specific communication of each cell subtype, including outgoing and incoming signalling, in the normal and RCT groups was also investigated. Outgoing signalling of NRG, ANGPTL and LAMNIN from the myonuclei was enhanced in the RCT group, whereas outgoing signalling of SEMA3, ANGPT and CADM was attenuated. The signalling pathways of SEMA3, HSPG, ADIPONECTIN, PDGF, VEGF and FGF in adipocytes and FAPs changed drastically, suggesting potential interactions between these two types of myonuclei and other resident cells (Figure S7B). Simultaneously, the incoming signalling pathways of PTPRM, BMP and CD99, and VISFATIN of myonuclei are altered significantly. Incoming signalling of BMP, THBS, NOTCH, ADIPONECTIN, VISFATIN and FGF showed different expression levels in adipocytes and FAPs in the RCT group (Figure S7B). Network analysis revealed that EGF signalling of FAPs as receivers was enhanced, and EGF signalling was reported to markedly activate asymmetric divisions of MuSCs, thereby increasing progenitor numbers, enhancing regeneration and restoring muscle strength.^49^, ^50^, ^51^ VEGF signalling in adipocytes as a sender was inhibited, indicating the reason for RCT‐induced vascular remodelling,^52^ and PTPRM signalling of type I and type II myonuclei as a sender and receiver was enhanced. In addition, BMP signalling was enhanced in endothelial cells and inhibited in type I myonuclei in the RCT group (Figure 7C,D). Previous studies have reported that BMP signalling transduces anabolic signals in myofibers.^53^, ^54^ ADIPONECTIN and FGF signalling were also observed in the normal group. ANGPTL signalling from adipocytes to FAPs was enhanced in the RCT group (Figure S7C).

### 2.8 Tissue distribution of distinct isoforms of myofibers and FAPs

**FIGURE 8:**
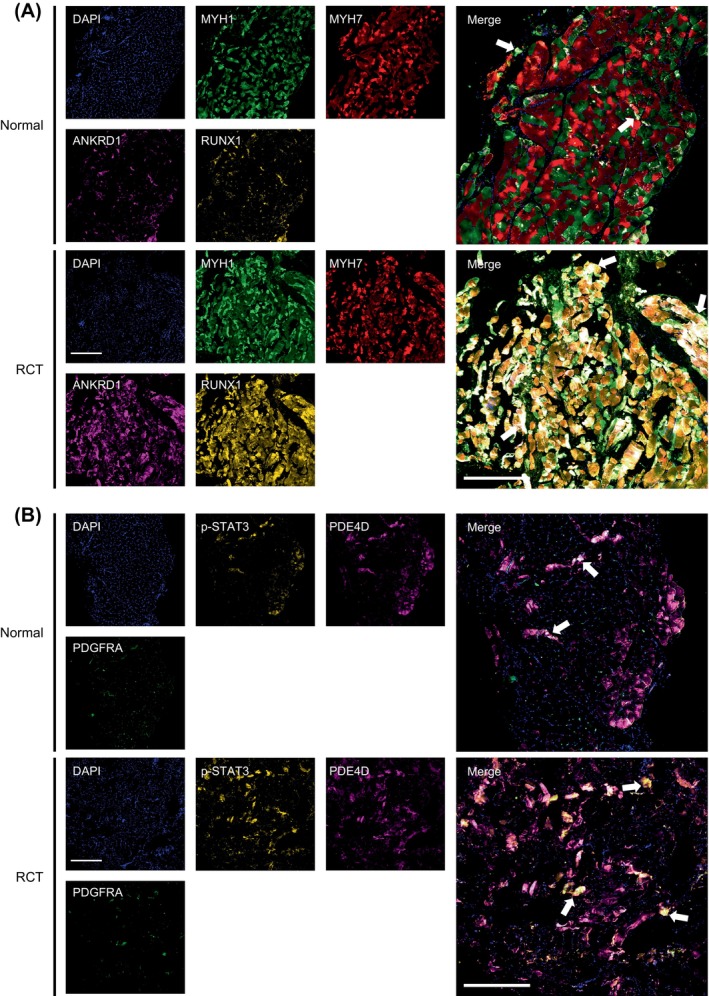
Anatomical localization of myofibers and fibro/adipogenic progenitors (FAPs) subsets. (A) Representative image showing multiplex immunofluorescence (mIF) staining of myonuclei subtypes in normal (upper panel) and rotator cuff tear (RCT) (lower panel) conditions. DAPI: blue, MYH1: green, MYH7: red, ANKRD1: magenta, RUNX1: yellow. Arrows: ANKRD1^+^/RUNX1^+^ muscle fibres. Scale = 400 μm. (B) Representative image showing mIF staining of FAPs subtypes in normal (upper panel) and RCT (lower panel) conditions. DAPI: blue, PDGFRA: green, PDE4D: magenta, p‐STAT3: yellow. Arrows: STAT3^+^/PDE4D^+^ FAPs. Scale = 400 μm.

Various types of muscle cells were histologically validated. In the RCT group, the supraspinatus muscle exhibited an increased presence of fat droplet structures compared to that in the normal group, accompanied by a disordered arrangement of muscle fibres and a reduction in volume (Figure S8A). Masson's trichrome staining indicated muscle degeneration after the RCT (Figure S8B). To corroborate the transcriptional heterogeneity of muscle cells under normal and RCT conditions, the multiplex immunofluorescence (mIF) results demonstrated significantly higher expression of ANKRD1 and RUNX1 in both type I (MYH7^+^) and type II (MYH1^+^) myofibers (Figure 8A, Figure S9A), which is consistent with our sequencing results. In addition, the co‐localisation of p‐STAT3 and PDGFRA was observed in the RCT group, indicating transcriptional changes in FAPs after RCT (Figure 8B, Figure S9B).

## 3 DISCUSSION

RCT is a severe musculoskeletal problem in which mechanical unloading induces the degeneration of the rotator cuff muscle, characterised by muscle atrophy, fatty infiltration and fibrosis.^6^ Currently, the molecular mechanisms and potential cell communication following RCT remain unclear, and effective treatments for post‐RCT muscle degeneration are yet to be established. In this study, we examined the phenotypic and transcriptomic alterations in myonuclei and other resident cells caused by RCT, exploring the intricate interplay among different cells to identify potential therapeutic targets and strategies for the treatment of RCT.

Skeletal muscles demonstrate significant adaptability in response to environmental changes, involving intricate processes such as protein synthesis and degradation, metabolic reprogramming, and tissue remodelling.^55^ In our snRNA‐seq analysis, we observed trajectory transitions from normal type IIx and IIa myonuclei to ANKRD1^+^ type II myonuclei after RCT and noted a shift from normal ATP2A2^+^ type I myonuclei to ANKRD1^+^ type I myonuclei. Interestingly, KEGG enrichment analysis demonstrated that the FoxO signalling pathway and insulin resistance were enriched in ANKRD1^+^ myonuclei, which have been shown to be related to muscle atrophy and adipocyte differentiation.^56^, ^57^ ANKRD1 is predominantly expressed in cardiac and foetal skeletal muscles, but is rarely detected in adult skeletal muscles.^58^, ^59^ Its expression in the skeletal muscles is induced under certain conditions, including denervation and muscle pathology.^60^, ^61^ ANKRD1 expression is induced in hypertrophic skeletal muscles and atrophic myofibers in patients with spinal muscular atrophy (SMA).^62^, ^63^ Our research suggests the active involvement of both fast‐ and slow‐twitch fibres in post‐RCT muscle degeneration, ANKRD1 may be crucial for unravelling the signalling pathways that govern skeletal muscle remodelling processes, including atrophy and hypertrophy.

In response to muscle injury, FAPs undergo activation, proliferation and expansion, creating a transiently favourable environment that promotes MuSCs‐mediated regeneration.^64^, ^65^, ^66^ FAPs have also been identified as a major source of infiltrating fibroblasts and adipocytes in degenerating dystrophic muscles.^67^, ^68^, ^69^, ^70^ Trajectory analysis revealed the transition from COL3A1^+^ and COL15A1^+^ FAPs to STAT3^+^ FAPs. Notably, the proportion of STAT3^+^ FAPs increased, which was accompanied by a decrease in the proportion of COL3A1^+^/COL15A1^+^ FAPs. A previous study showed that denervation leads to the progressive accumulation of FAPs with persistent STAT3 activation, contributing to muscle atrophy and fibrosis.^71^ During the regeneration process, FAPs play a crucial role in transient fibrosis necessary for maintaining the structural integrity of the regenerated muscle. As the regeneration process concludes, the resolution of fibrosis involves a decrease in the number of FAPs and dampening of the expression of profibrotic genes.^72^ Persistent or unresolved fibrosis is known to impair muscle function and is a characteristic feature of diseased muscle.^73^, ^74^ Interventions targeting the formation of the STAT3^+^ FAPs sub‐cluster may present novel therapeutic targets and strategies for addressing muscle atrophy and fat infiltration following RCT.

We also determined the GRNs of different cell types under normal and RCT conditions. Muscle atrophy is mediated by a group of atrophy‐related genes, such as KLF5, KLF10, STAT3 and BHLHE40.^27^, ^28^, ^30^, ^75^, ^76^, ^77^, ^78^ KLF5 and KLF10 expression significantly increases with muscle wasting and correlates positively with the expression of the pro‐atrophy muscle‐specific ubiquitin ligase genes FBXO32 and TRIM63.^27^, ^28^ In patients with sepsis, the IL‐6/JAK/STAT3 pathway is highly up‐regulated and associated with muscle atrophy.^76^ In addition, JAK/STAT signalling is highly expressed in old mice and its inhibition leads to the recovery of muscle function.^79^ TEAD4, FOS and MYC are essential for embryonic skeletal muscle development,^80^, ^81^, ^82^ activation of these regulons in RCT implicates the activation of an early developmental program in the pathogenesis of muscle atrophy. TBX15 and CREB1 are rarely expressed in ANKRD1^+^ myonuclei, and studies have shown that they control skeletal muscle fibre types and muscle metabolism.^83^, ^84^ Increasing evidence has highlighted the significant role of oxidative stress in the pathophysiology of various muscle disorders, both in early and adult‐onset cases,^85^ In ANKRD1^+^ myofibers post‐RCT, there was an elevation in NFE2L2, RB1 and ZEB1, suggested a potential promotion of an adaptive antioxidant response during muscle atrophy. This response may protect myofibers from mitochondrial damage and excessive levels of reactive oxygen species (ROS).^86^ However, the effects of the other regulons have not been investigated.

Our findings provide comprehensive insights into the transcriptome repertoire of muscle cells and other resident cells, including type I and type II myonuclei, and other muscle‐resident cells in the supraspinatus before and after RCT. These results also highlight the potential cellular and transcriptional targets for attenuating muscle degeneration after RCT.

## 4 METHODS

### 4.1 Patients

This study was approved by the Institutional Review Board of the Jinling Hospital (2023DZKY‐090‐02) and adhered to the Declaration of Helsinki. Written informed consent for this study was obtained from all patients. In total, the RCT group consisted of three patients with RCTs confirmed using magnetic resonance imaging (MRI) and arthroscopy. The normal group included three patients with MRI and arthroscopically confirmed habitual shoulder dislocation. For the validation assays, another five patients with RCT and five patients with habitual shoulder dislocation were included. Inclusion criteria: the Goutallier scores of the RCT patients were ≥ 2 and combined with persistent non‐relief shoulder pain, excluding other basic diseases and history of shoulder trauma.^87^ Patients in the normal group had habitual dislocation of the shoulder joint due to labial lesions, and those with congenital articular sac relaxation, basic disease or rotator cuff injury were excluded.

### 4.2 Sample collection

Tissue samples were obtained from the supraspinatus muscle and assessed for the presence of fatty accumulation. Similar to a previous study, all biopsy specimens comprised identifiable muscle fibres located medial to the musculotendinous junction with dimensions of <3 × 3 mm.^88^ The scapular spine was skeletonised to distinguish the supraspinatus from the infraspinatus, using a posterolateral portal for visualisation and an anterolateral portal for the operation. Care was taken to ensure a clear visualisation of the muscle medial to the musculotendinous junction. Utilising a 2.4‐mm arthroscopic biter, a muscle biopsy specimen was obtained and subsequently stored at −80°C for subsequent analyses.

### 4.3 Muscle nuclei isolation

Muscle nuclei were isolated using nuclei EZ lysis buffer (NUC‐101, Sigma‐Aldrich) supplemented with a protease inhibitor (5892791001; Roche) and an RNase inhibitor (N2615; Promega and AM2696; Life Technologies). Muscle samples were cut into 1 mm pieces and homogenised using a Dounce homogeniser (885302‐0002; Kimble Chase) in 2 mL of ice‐cold nuclei EZ lysis buffer. They were then incubated on ice for 5 min with an additional 2 mL of lysis buffer, ground with a Dounce (Sigma), and gently resuspended in a pipette. Muscles were incubated on ice for 6 min, and then 2 mL of ice‐cold 4% bovine serum albumin (BSA), resuspended in a Pasteur pipette, and the reaction was stopped. The homogenate was sequentially centrifuged at 300 × g for 5 min at 4°C, 2 mL of lysis buffer and 4% BSA were added and resuspended. After incubation on ice for 3 min and de‐fragmentation with Debris Removal Solution (Miltenyi), the pellet was resuspended, washed with 4 mL of buffer, and then incubated on ice for 5 min. After another centrifugation, the pellet was resuspended in Nuclei Suspension Buffer (0.07% BSA albumin and 0.1% RNase inhibitor). Subsequently, it was filtered through a 20‐mm cell strainer (43‐50020‐50, pluriSelect), and the count was determined using an Automated Cell Counter (Haemocytometer/Countess II). The concentration was then adjusted to 700–1200 cells/μL.

### 4.4 Chromium 10× genomics library and sequencing

Single‐nucleus suspensions were introduced into the 10× chromium system to capture individual cells in accordance with the manufacturer's guidelines for the 10× Genomics Chromium Single‐Cell 3 kit (version 3.1). The subsequent steps involving cDNA amplification and library construction were performed according to standard protocols. The resulting libraries were sequenced on an Illumina HiSeq 6000 sequencing system (paired‐end multiplexing run, 150 bp) by LC‐Bio Technology Co., Ltd. (Hangzhou, China), ensuring a minimum depth of 20,000 reads per cell. All cells were filtered under quality control conditions (the expression of all genes was detected in at least three cells, with more than 200 genes expressed in a single cell, the ratio of mitochondrial gene expression was <25%, and multiple cells were removed using the DoubletFinder package).

### 4.5 Bioinformatics analysis

The sequencing results were demultiplexed and converted to the FASTQ format using Illumina bcl2fastq software (version 5.01). Next, sample demultiplexing, barcode processing and single‐cell 3′ gene counting was performed using the Cell Ranger pipeline (version 3.1.0) (https://support.10×genomics.com/single‐cell‐gene‐expression/software/pipelines/latest/what‐is‐cell‐ranger). snRNA‐seq data were aligned to the GRCh38 reference genome. A total of 20,850 single nuclei, captured from three patients with habitual shoulder dislocation and three patients with RCT, were processed using the 10× Genomics Chromium Single‐Cell 3′ solution.

The cell range output was then imported into Seurat (version 4.1.0) for dimensional reduction, clustering, and analysis of the snRNA‐seq data. After quality control, 16,893 cells passed the threshold, removing genes expressed in fewer than three cells (default parameters: one cell), considering a low cut‐off of fewer than 500 genes expressed per cell. In addition, UMI counts less than 500 and the percentage of mitochondrial DNA‐derived gene expression less than 25% were used as quality control criteria. To visually represent the data, we further reduced the dimensionality of all 16,893 cells using Seurat and employed uniform manifold approximation and projection (UMAP) to project the cells into a 2D space. Normalisation was performed using the LogNormalize method with the ‘Normalization’ function of Seurat software to calculate the expression values of genes. Principal component analysis (PCA) was performed on normalised expression values. Among the principal components (PCs), the top 10 were selected for subsequent clustering and UMAP analysis. To discern distinct cellular populations, a weighted shared nearest neighbour (SNN) graph‐based clustering method was used. Subsequently, the marker genes characterising each cluster were identified using the Wilcoxon rank‐sum test. The FindAllMarkers function in Seurat was used to facilitate this process by selecting genes expressed in >10% of the cells within a cluster with an average log(Fold Change) of 0.26.

SCENIC is a computational method for constructing regulatory networks and identifying distinct cellular states from snRNA‐seq data. To assess the differences between cell clusters, we measured variations in transcription factors or their target genes. SCENIC analysis of all snRNA‐seq data were performed using pySCENIC (v0.12.1) with the database for motif enrichment set to mc_v10_clust.

Metadata of the Seurat object, expression matrix, and UMAP results were loaded into Monocle3 (version 1.3.4) to generate the CellDataSet object. The CellDataSet object was preprocessed using the preprocess_cds function. Subsequently, trajectory analysis was performed using the learn_graph function to identify the trajectory, and order_cells was used to calculate the position of each cell in the proposed time.

CellChat (v1.6.1) was used to generate CellChat objects using metadata from the Seurat objects and normalised expression matrices. Subsequently, the identifyOverExpressedGenes and identifyOverExpressedInteractions functions were used to confirm highly expressed genes, receptors and ligands. Next, the computeCommunProb function was used to calculate the communication probability and infer the CellChat network. Finally, the communication probabilities and integration results at the signalling pathway level were calculated using the computeCommunProbPathway function and the aggregateNet function, respectively.

### 4.6 Histological analysis

Human supraspinatus muscle specimens were fixed in 4% paraformaldehyde (PFA) for 48 h. Subsequently, they were embedded in paraffin and sectioned into 5‐mm‐thick slices for haematoxylin and eosin (H&E) and Masson staining. For H&E staining, paraffin sections were fixed in 4% PFA, washed in phosphate‐buffered saline (PBS), and stained with haematoxylin for 4 min, followed by eosin for 6 min. To assess fibrosis and fatty infiltration, staining was performed using a Masson's trichrome kit (G1346, Solarbio) according to the manufacturer's instructions.

### 4.7 mIF staining

Muscle sections were de‐paraffinised. After antigen retrieval, the sections were blocked with 3% H_2_O_2_ and 2% BSA. Different primary antibodies, anti‐myosin (Skeletal, Slow) (M8421, Sigma‐Aldrich), anti‐myosin (Skeletal, Fast) (M4276, Sigma‐Aldrich), anti‐ANKRD1 (11427‐1‐AP, Proteintech), anti‐AML1(RUNX1) (4336T, Cell Signalling Technology), anti‐PDGF receptor α (D1E1E) (3174S, Cell Signalling Technology), anti‐phospho‐stat3 (Tyr705) (9145S, Cell Signalling Technology) and anti‐PDE4D (12918‐1‐AP, Proteintech) were sequentially used, followed by horseradish peroxidase conjugated secondary antibody incubation (F2761, F2765, T2769 and PA1‐28565; Thermo Fisher Scientific) and tyramide signal amplification (TSA) ([FITC‐TSA, CY3‐TSA, 594‐TSA and CY5‐TSA YB007, YOBIBIO]). After labelling with human antigens, the nuclei were stained with 4′,6‐diamidino‐2‐phenylindole (DAPI; UBI5010, YOBIBIO), and an antifade mounting medium was applied. Stained slides were scanned using a Pannoramic MIDI Scanner (3D HISTECH, Hungary) to obtain multispectral images. DAPI glows blue upon ultraviolet (UV) excitation at 330–380 nm with an emission wavelength of 420 nm in the fluorescence spectra. FITC glows green at excitation and emission wavelengths of 465–495 nm and 515–555 nm, respectively. CY3 glows red at excitation and emission wavelengths of 510–560 nm and 590 nm, respectively. A total of 594 glows yellow at excitation and emission wavelengths of 576–596 and 618–638 nm, respectively. CY5 glows magenta at excitation and emission wavelengths of 608–648 nm and 672–712 nm, respectively. Multispectral images were analysed, and positive cells were quantified at the single‐cell level using the Caseviewer (version 2.3) image analysis software.

### 4.8 Statistics

Comparisons between two groups were performed using the Student's *t*‐test or Wilcoxon rank‐sum test. Comparisons of more than two groups were performed using the Kruskal–Wallis test. Statistical significance was set at *p* < 0.05.

## AUTHOR CONTRIBUTIONS

Ziying Sun and Xi Cheng designed and supervised the study, prepared the figures and wrote the manuscript. Ziying Sun, Zheng Wang, Zhihao Lu, Yuan Liu and Hanwen Zhang performed the computational analyses and assisted in data analysis in discussions with Nirong Bao, Zhongyang Lv and Xi Cheng. Nirong Bao, Jia Meng and Xingyun Wang also critically reviewed the manuscript. Hong Qian, Chengteng Lai, Yang Wang, Wenshuang Sun, Jintao Lin and Xiaojiang Yang performed patient sample processing and histological analysis. Nirong Bao, Hong Qian, Chenfeng Qiao and Tao Yuan gathered patients' consent, provided the samples and clinical data.

## FUNDING INFORMATION

This research was funded by the Scientific Research Project of Jiangsu Commission of Health (LKZ2022011), Jiangsu Funding Program for Excellent Postdoctoral Talent (2022ZB744, 2022ZB753), China Postdoctoral Science Foundation (2022M723892, 2023M734292).

## CONFLICT OF INTEREST STATEMENT

The authors declare that they have no conflict of interest.

## Supporting information


**Data S1.** Supporting Information.

## Data Availability

The raw sequence data reported in this paper have been deposited in the Genome Sequence Archive (Genomics, Proteomics & Bioinformatics 2021) in National Genomics Data Centre (Nucleic Acids Res 2022), China National Center for Bioinformation/Beijing Institute of Genomics, Chinese Academy of Sciences (GSA‐Human: HRA006207), that are publicly accessible at https://ngdc.cncb.ac.cn/gsa-human. All data needed to evaluate the conclusions in the paper are available in the NGDC or present in the paper and/or the Supplementary Materials. Additional data related to this paper may be requested from the authors.
