# Supplementary material for: Single‐nucleus transcriptomics reveals subsets of degenerative myonuclei after rotator cuff tear‐induced muscle atrophy
Source: Cell Prolif. 2024 Oct 22;58(3):e13763. doi: 10.1111/cpr.13763 (PMC11882757; doi:10.1111/cpr.13763)
Supplement: Supplementary file 1 — Data S1. Supporting Information. [file CPR-58-e13763-s001.docx]

**Supplementary Information**

**Single-nucleus RNA-seq identifies transcriptional heterogeneity of the supraspinatus muscle after rotator cuff tear**

Sun et al.

Supplementary Figure 1


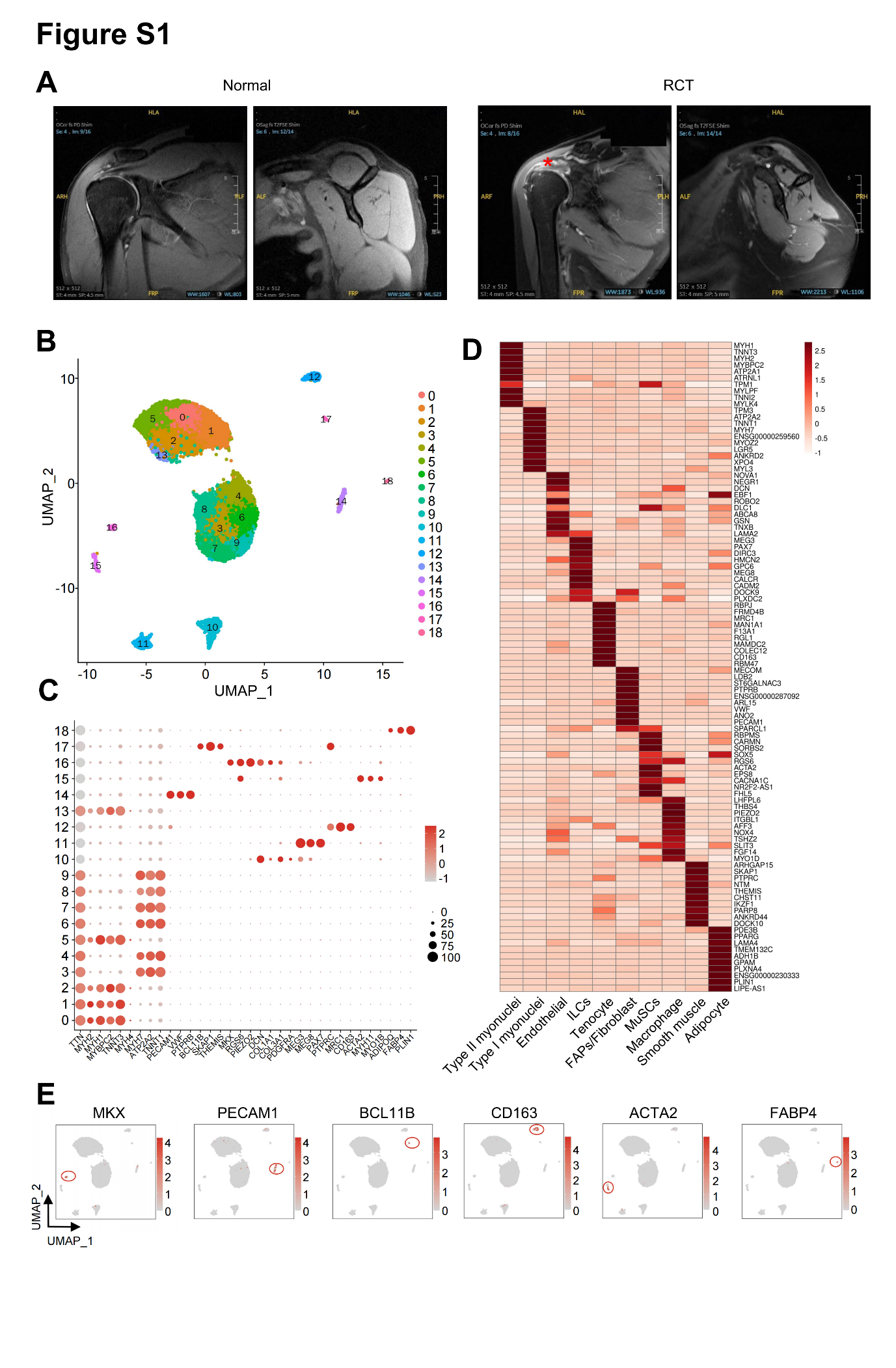


Supplementary Figure 1. Unsupervised classification of nucleus/cell types of supraspinatus muscles in normal and rotator cuff tear (RCT) patients. A. Representative magnetic resonance imaging (MRI) images of the coronal and oblique sagittal views of the shoulder after rotator cuff tear. B. Uniform manifold approximation and projection (UMAP) plot displaying the unsupervised clusters in Seurat (resolution=1.2). Each cluster is colour coded. C. Dot plot showing the expression of cell identities in unsupervised clusters. Dot size represents the percentage of nuclei expressing a gene. The colour intensity of dot indicates gene expression level and the colour scale indicates the expression level. D. Heatmap showing the top 10 signature genes of each cluster, identified by Seurat “FindAllMarkers” function across the whole dataset (logfc>0.26, min. pct>0.1). E. UMAP plot displaying the cell identity of specific nuclei cluster.

Supplementary Figure 2


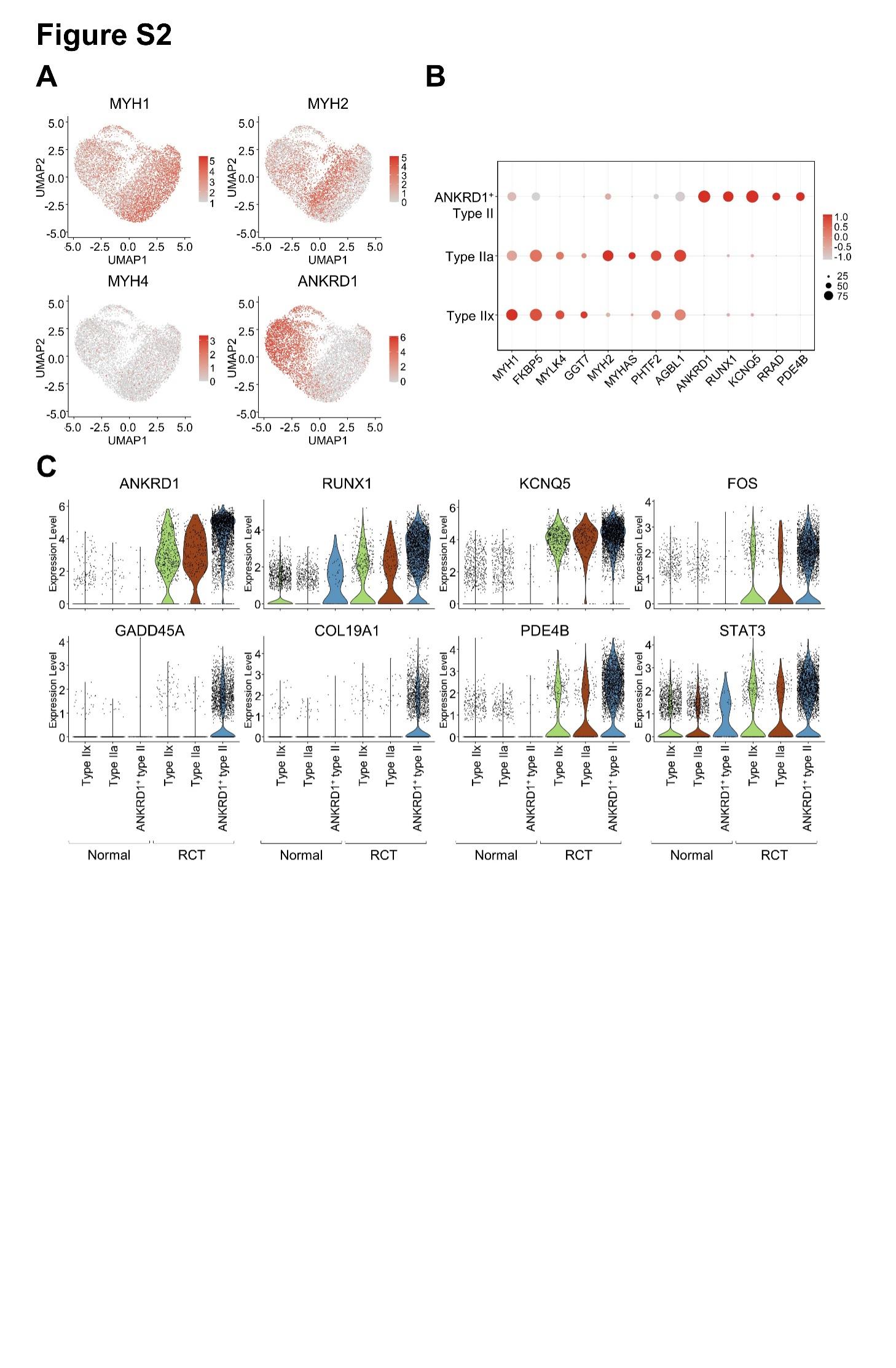


Supplementary Figure 2. Identities expression and distribution of each subtype of type II myonuclei. A. UMAP plot displaying the cell identity of each identified subtype of type II myonuclei, including MYH2, MYH2, MYH4, ANKRD1, etc. B. Dot plot showing the expression of cell identities in each identified subtype. Dot size represents the percentage of nuclei expressing the specific gene. The colour intensity of dot indicates gene expression level and the colour scale indicates the expression level. C. Violin plot showing comparison of RCT-induced gene expression in different type of type II myonuclei.

Supplementary Figure 3


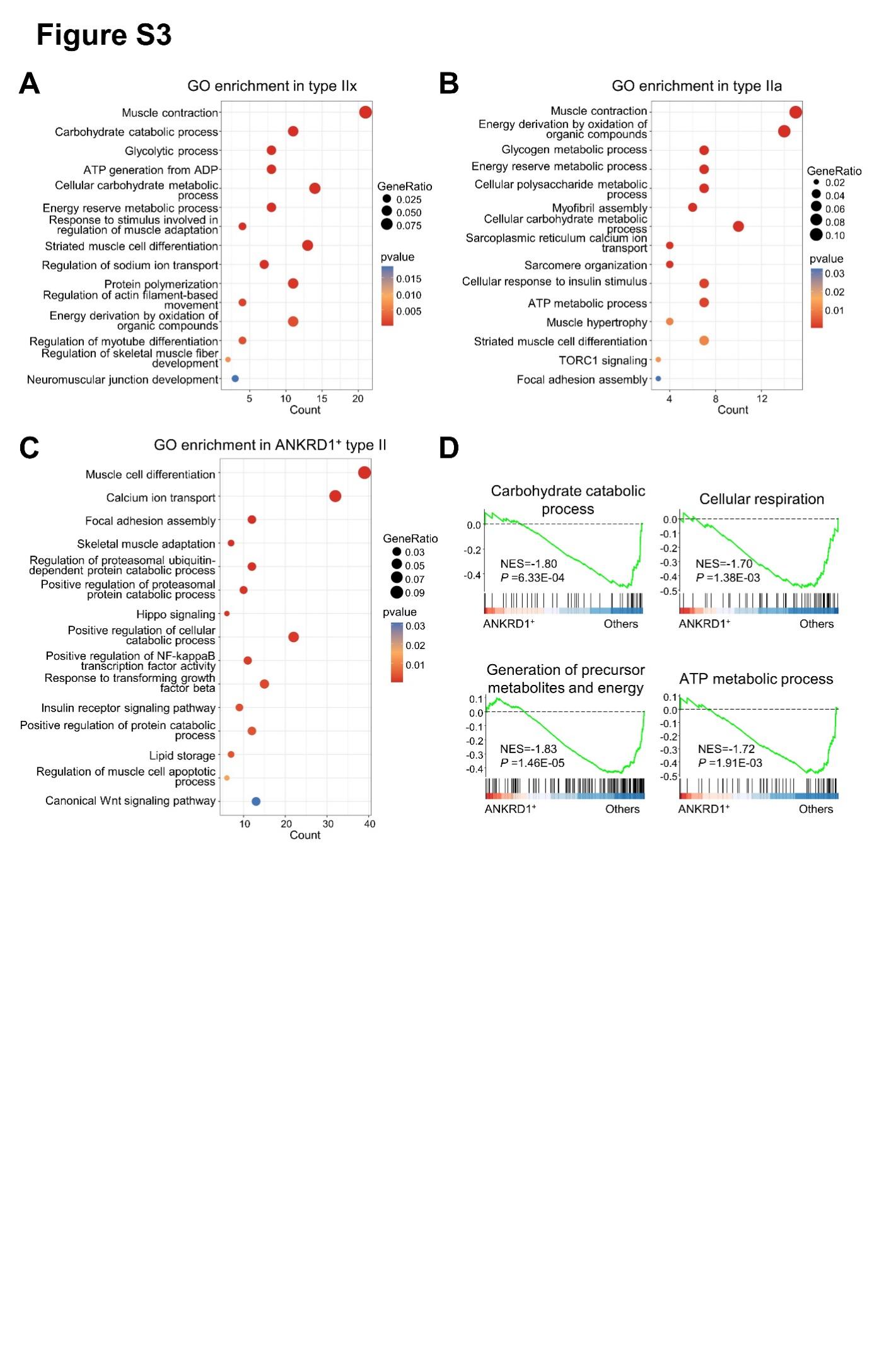


Supplementary Figure 3. Gene ontology (GO) enrichment analysis of each subtype of type II myonuclei. A. Enriched gene ontology biological process (GOBP) terms (*P* < 0.05) in type IIx myonuclei. The colour scale indicates the significance level of enrichment (*P* value). Dot size represents gene ratio in the term. B. Enriched GOBP terms (*P* < 0.05) in type IIa myonuclei. The colour scale indicates the significance level of enrichment (*P* value). Dot size represents gene ratio in the term. C. Enriched GOBP terms (*P* < 0.05) in ANKRD1^+^ type II myonuclei. The colour scale indicates the significance level of enrichment (*P* value). Dot size represents gene ratio in the term. D. Gene set enrichment analysis (GSEA) plots showing value of enrichment score (ES) of the significant enriched GOBP gene sets in ANKRD1^+^ type II myonuclei. A negative ES indicates enrichment in other type II myonuclei but down-regulated in ANKRD1^+^.

Supplementary Figure 4


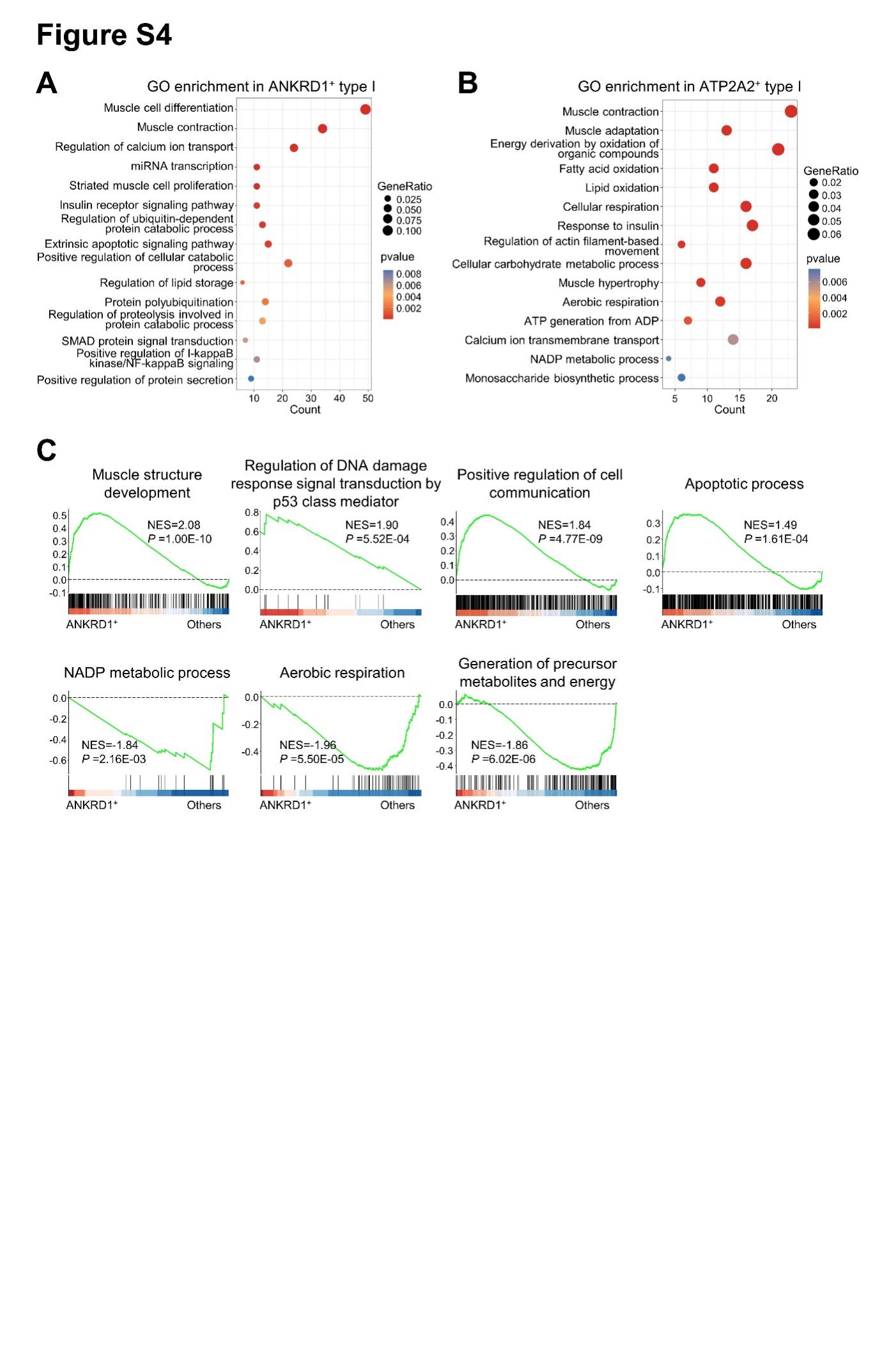


Supplementary Figure 4. GO enrichment analysis of each subtype of type I myonuclei. A. Enriched GOBP terms (*P* < 0.01) in ANKRD1^+^ type I myonuclei. The colour scale indicates the significance level of enrichment (*P* value). Dot size represents gene ratio in the term. B. Enriched GOBP terms (*P* < 0.01) in ATP2A2^+^ type I myonuclei. The colour scale indicates the significance level of enrichment (*P* value). Dot size represents gene ratio in the term. C. GSEA plots showing ES of the significant enriched GOBP gene sets in ANKRD1^+^ type I myonuclei. A positive value of ES indicates enriched in ANKRD1^+^ type I myonuclei, and a negative value indicates enriched in other type I myonuclei but down-regulated in ANKRD1^+^.

Supplementary Figure 5


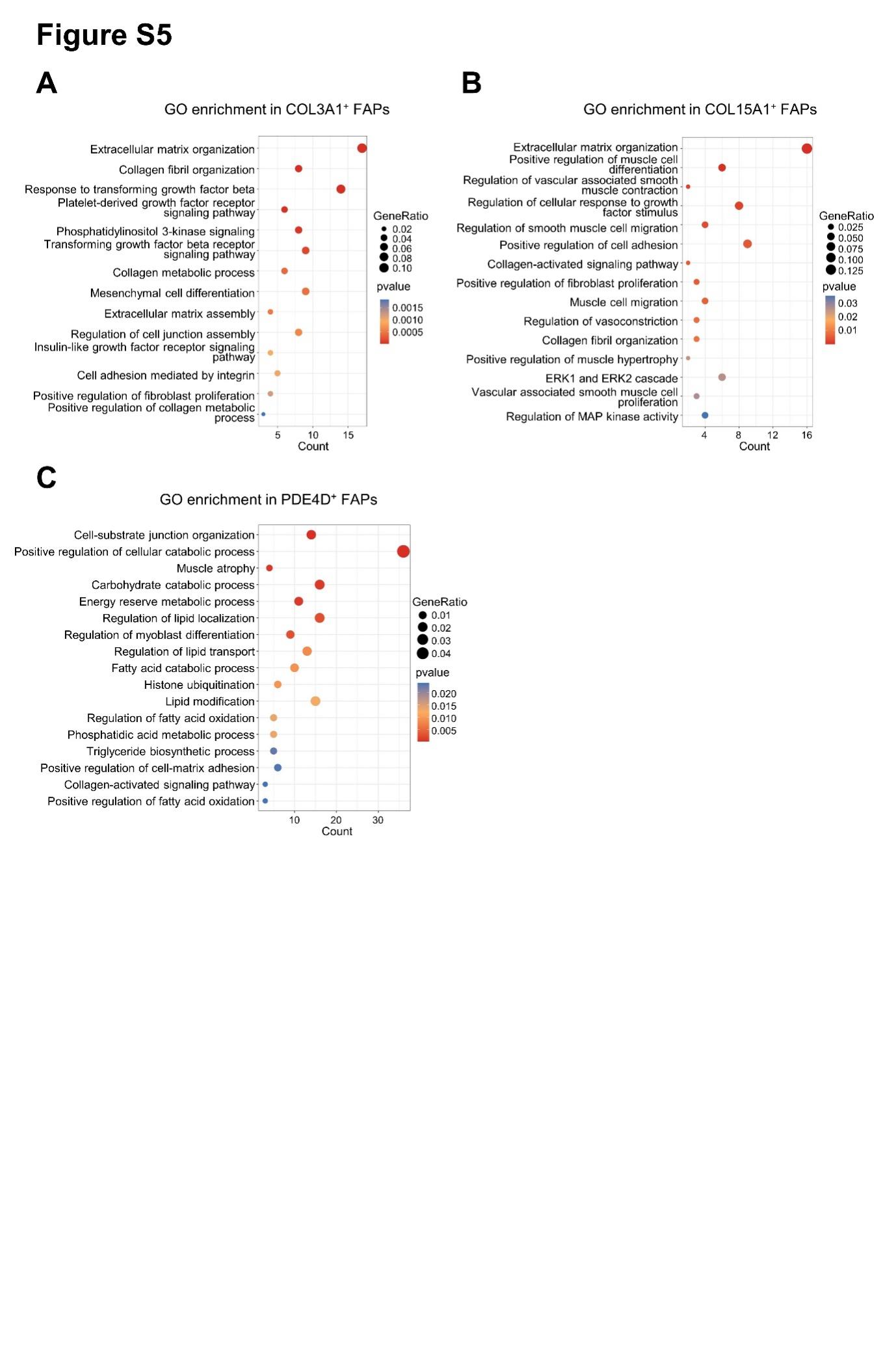


Supplementary Figure 5. RCT induced changes of GO enrichment in FAPs. A. Enriched GOBP terms (*P* < 0.01) in COL3A1^+^ FAPs. The colour scale indicates the significance level of enrichment (*P* value). Dot size represents gene ratio in the term. B. Enriched GOBP terms (*P* < 0.05) in COL15A1^+^ FAPs. The colour scale indicates the significance level of enrichment (*P* value). Dot size represents gene ratio in the term. C. Enriched GOBP terms (*P* < 0.05) in PDE4B^+^ FAPs. The colour scale indicates the significance level of enrichment (*P* value). Dot size represents gene ratio in the term.

Supplementary Figure 6


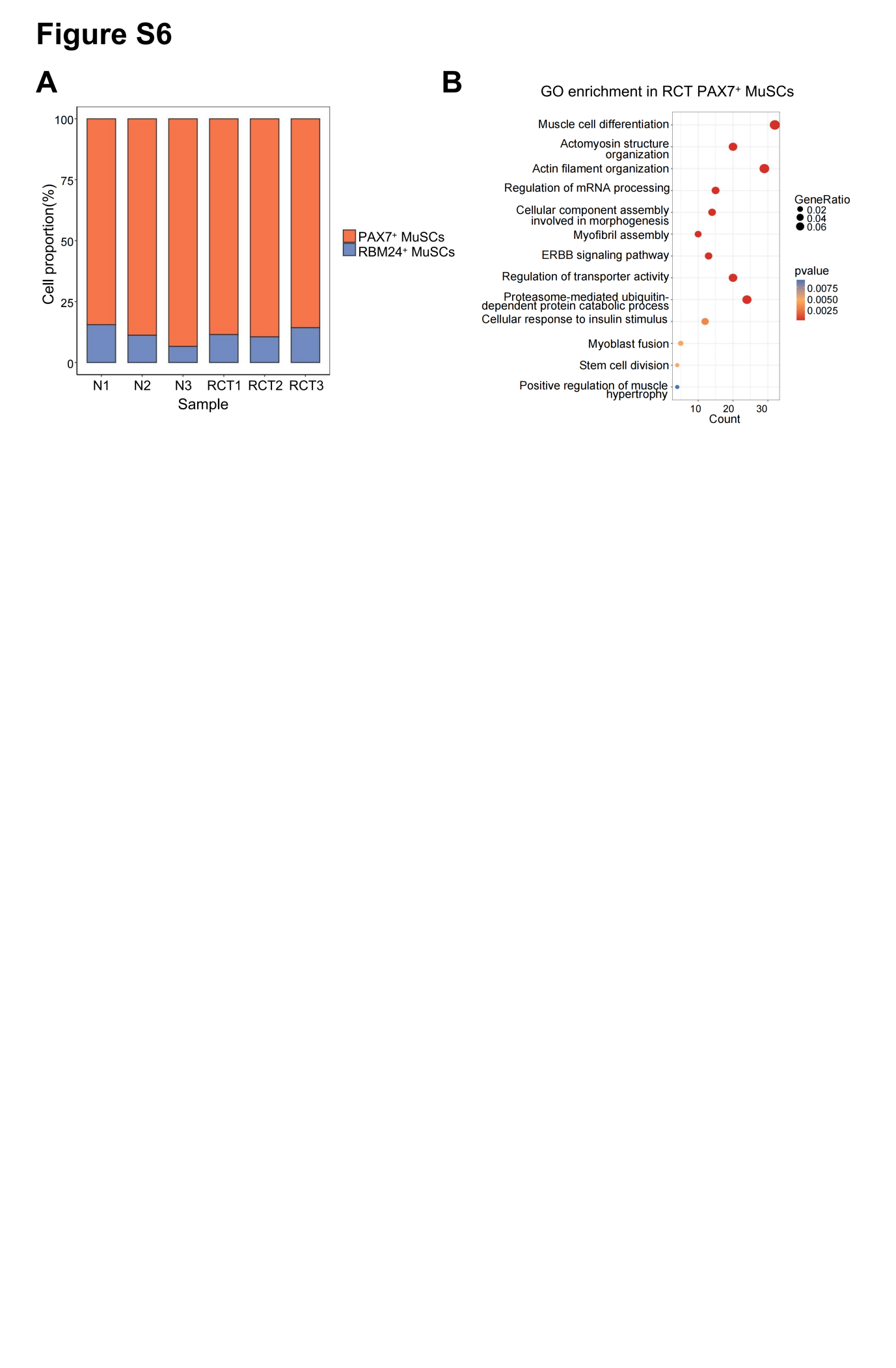


Supplementary Figure 6. Cell proportional distribution and functional enrichment of MuSC. A. Proportion of different MuSCs subtypes in normal and RCT conditions. B. Enriched GOBP pathways (*P* < 0.01) of PAX7^+^ MuSCs in RCT group. The colour scale indicates the significance level of enrichment (*P* value). Dot size represents gene ratio in the pathway.

Supplementary Figure 7


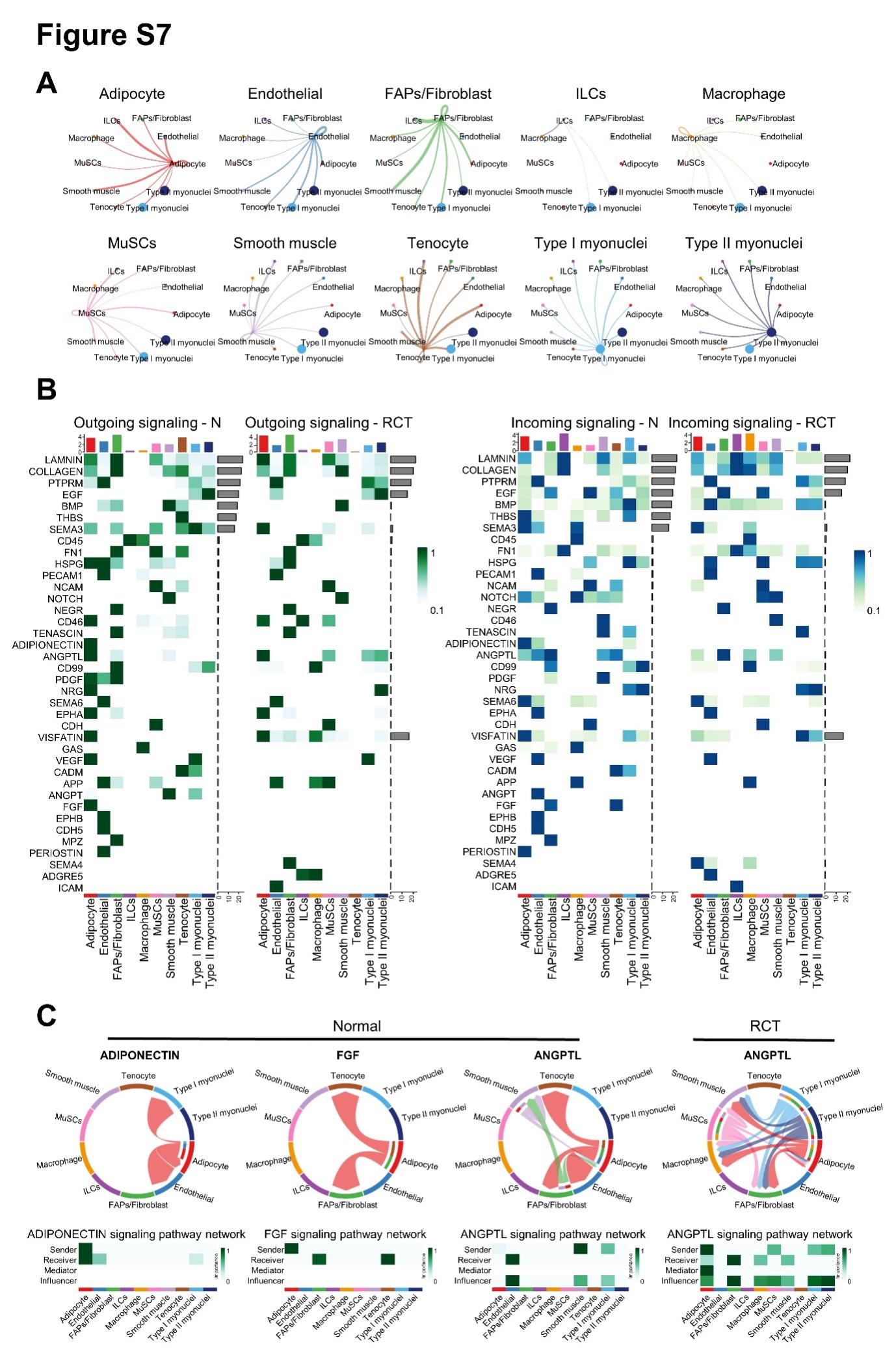


Supplementary Figure 7. Cell-cell communications in supraspinatus muscle mediated by ligand-receptor (L-R) interactions. A. Chrod plot displaying intercellular ligand-receptor (L-R) interactions between each cluster in supraspinatus muscles. B. Heatmap of the CellChat signaling in each nuclear cluster in normal and RCT muscles, including the outgoing signaling patterns (left panel) and the incoming signaling patterns (right panel). A gradient of white to dark green/blue indicates low to high expression weight value in the heatmap. C. Chord diagram inferred the ADIPONECTIN, FGF, ANGPTL signaling in normal muscles and ANGPTL signaling in RCT muscles with CellChat. The size of the width of various colours in the periphery indicates probability/intensity value of interaction (intensity is the sum of probability values). Heatmaps quantify the role of each cluster as a sender, receiver, mediator, and influencer.

Supplementary Figure 8


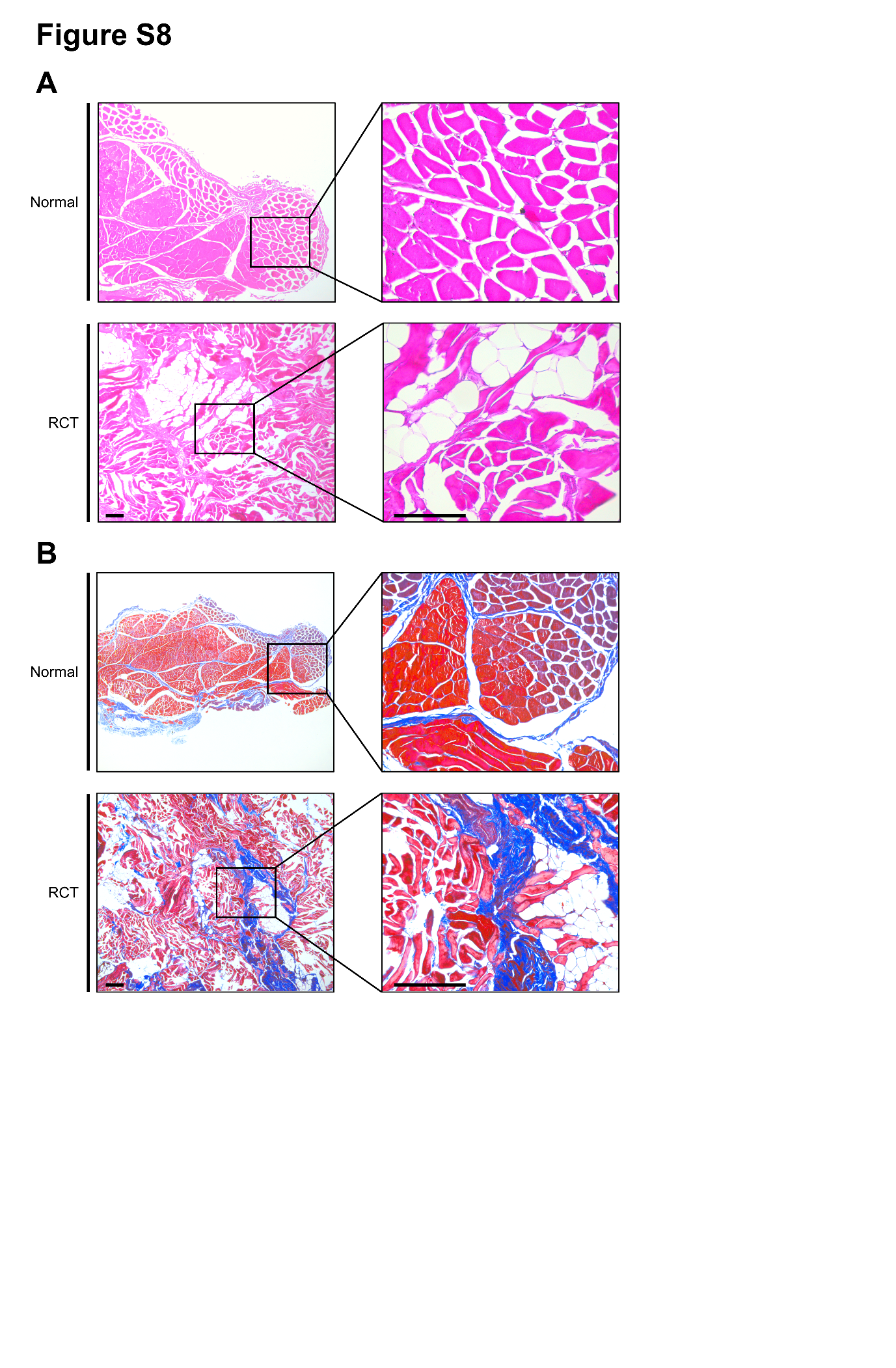


Supplementary Figure 8. Histological staining of supraspinatus muscles in normal and RCT conditions. A. Typical image of hematoxylin-eosin (H&E) staining of supraspinatus muscles in normal (upper panel) and RCT (lower panel) conditions. Scale = 100 µm. B. Typical image of Masson staining of supraspinatus muscles in normal (upper panel) and RCT (lower panel) conditions. Blue‐collagen, red‐muscle, and purple‐nucleus. Scale = 200 µm.

Supplementary Figure 9


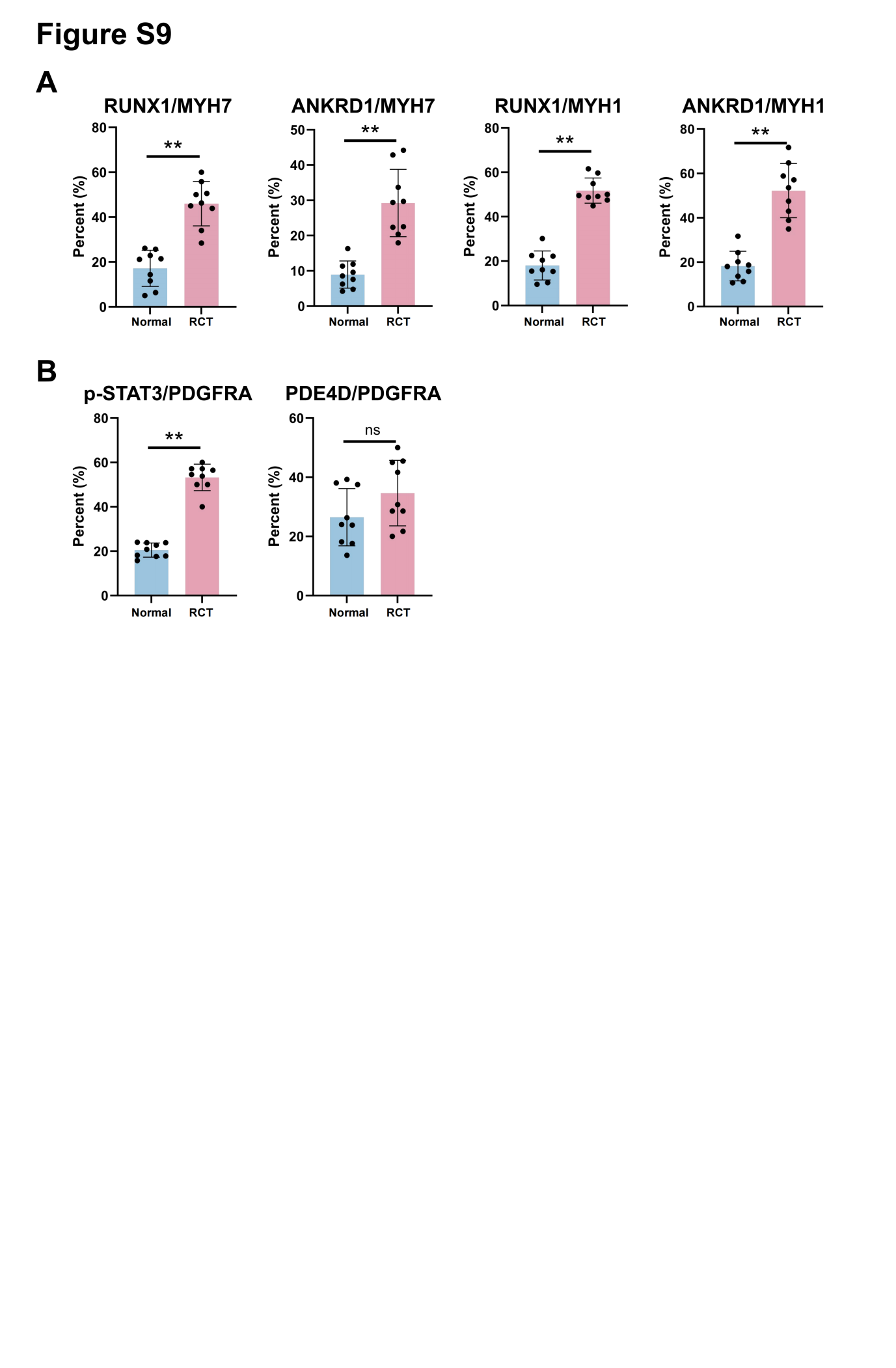


Supplementary Figure 9. Quantitative analysis of multiplex immunofluorescence (mIF) staining in Figure 8. A. The proportion of ANKRD1^+^ type II myonuclei and type I myonuclei after RCT was significantly higher than that in the Normal group. B. STAT3^+^ FAPs were significantly higher in the RCT group compared to the Normal group. Two-tailed unpaired t-test. Data are shown as mean ± SD. **P < 0.01.
